# Synthesis, Evaluation, and Mechanism Study of New Tepotinib Derivatives as Antiproliferative Agents

**DOI:** 10.3390/molecules24061173

**Published:** 2019-03-25

**Authors:** Niu-niu Zhang, Bai-jiao An, Yan Zhou, Xing-shu Li, Ming Yan

**Affiliations:** Institute of Drug Synthesis and Pharmaceutical Process, School of Pharmaceutical Sciences, Sun Yat-sen University, Guangzhou 510006, China; zhangniuniuu@yeah.net (N.-n.Z.); 15521085401@163.com (B.-j.A.); zhouy598@mail2.sysu.edu.cn (Y.Z.)

**Keywords:** antiproliferative agents, c-Met inhibitor, 97H cells, low toxicity

## Abstract

Inspired by the potent inhibition activity of the c-Met (mesenchymal−epithelial transition factor) inhibitor Tepotinib, a series of new Tepotinib derivatives were synthesized and evaluated for their ability to act as antiproliferative agents to find the leading compounds with good activity and limited side effects. Among them, compound **31e** exhibited potent antiproliferative activity (IC_50_ (50% inhibitory concentration) = 0.026 μΜ) against hepatic carcinoma 97H (human liver cancer cell) cells and, importantly, had very low inhibitory activity against normal cells. A mechanism study demonstrated that **31e** induced G1 phase (First growth phase or G indicating gap) arrest, inhibited the phosphorylation of c-Met and its downstream signaling component, Akt (Protein Kinase B), and also inhibited the migration of hepatic carcinoma 97H cells.

## 1. Introduction

**Figure 1:**
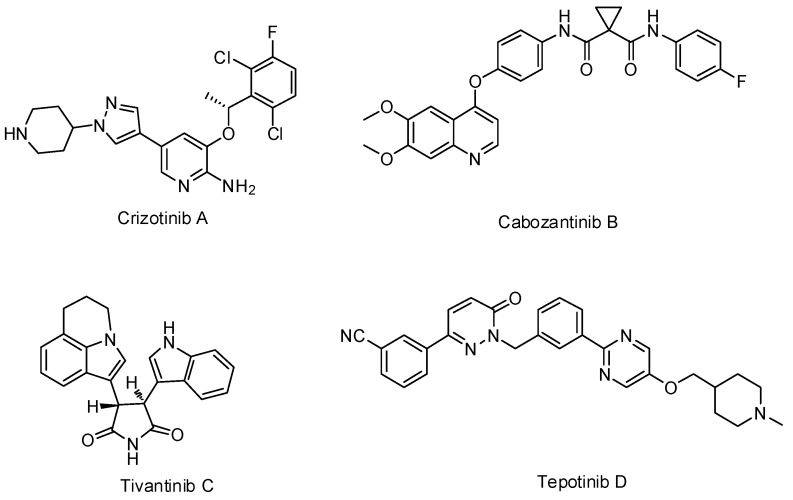
The representative c-Met kinase inhibitors of different structural types.

Cancer is a major disease that seriously threatens human health. According to statistics from the World Health Organization (WHO), an estimated 9.6 million patients died from cancer in 2018 [1]. Therefore, the development of effective anti-tumor drugs is an important task for medicinal chemists. Mesenchymal−epithelial transition factor (c-Met), the high affinity receptor for hepatocyte growth factor (HGF), is a unique subfamily of receptor tyrosine kinases (RTKs) [2]. Physiologically, HGF/c-Met signaling plays important roles in cell growth, survival, motility, and morphogenesis [3]. However, aberrant c-Met activation has been observed in a wide variety of human cancers, including liver and lung cancer, as a consequence of gene amplification or rearrangement, transcriptional regulation, as well as autocrine or paracrine ligand stimulation [4,5,6,7,8,9]. Due to its status as a proto-oncogene and the correlation of its dysregulation with a poor prognosis, c-Met is becoming a promising target for cancer therapy [10]. The present c-Met kinase inhibitors are divided into three groups according to their binding modes and structural features. Type I c-Met kinase inhibitors, such as Crizotinib A, are adenosine triphosphate (ATP)-competitive inhibitors with a U-shaped binding mode at the ATP binding site (Figure 1) [11,12]. Type II c-Met kinase inhibitors are usually multi-targeted agents (for example, Cabozantinib B) (Figure 1) [13]. Type I inhibitors are generally described as specific for c-Met kinase and more selective than class II inhibitors. However, they have limited activity against the Tyr1230His mutation that is present in certain human tumors [4]. Type III inhibitors include other atypical c-Met kinase inhibitors, such as Tivantinib C (Figure 1) [14]. 

In 2015, Dorsch et al. reported optimized pyridazinones as being potent and selective c-Met kinase inhibitors [15]. We found that the optimal compound, Tepotinib D (Figure 1), displays excellent in vitro potency and in vivo anti-tumor efficacy at low doses. According to the co-crystal structure of Tepotinib D with c-Met, it belongs to the type I c-Met kinase inhibitors. The review by Manjunath D. Ghate et al. described the recent advances of small molecule c-Met kinase inhibitors, including several pyridazinone derivatives, but the similar structures of our compounds have not been reported before [16]. Inspired by the interesting scaffold of Tepotinib, we decided to synthesize and evaluate new Tepotinib derivatives to find leading compounds with good activity and limited side effects.

**Figure 2:**
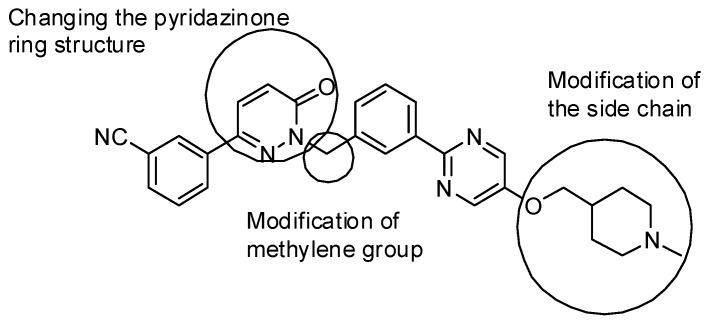
Design of new Tepotinib derivatives.

Our design is shown in Figure 2 and includes the following steps: (a) Changing the position of nitrogen on the pyridazinone ring to provide pyrazin-2(1*H*)-one and pyrimidin-2(1*H*)-one derivatives for the study of the structure–activity relationships; (b) evaluating the anti-tumor activity of the derivatives with or without alkyl substitution on the methylene group; (c) examining the effect of fluorine substitution on pyridin-2(1*H*)-one on the anti-tumor activity; and (d) investigating the effect of side chains on the activity. Herein, we reported the synthesis, evaluation, and optimization of new Tepotinib derivatives as anti-tumor agents.

## 2. Results

### 2.1. Chemistry

**Scheme 1:**
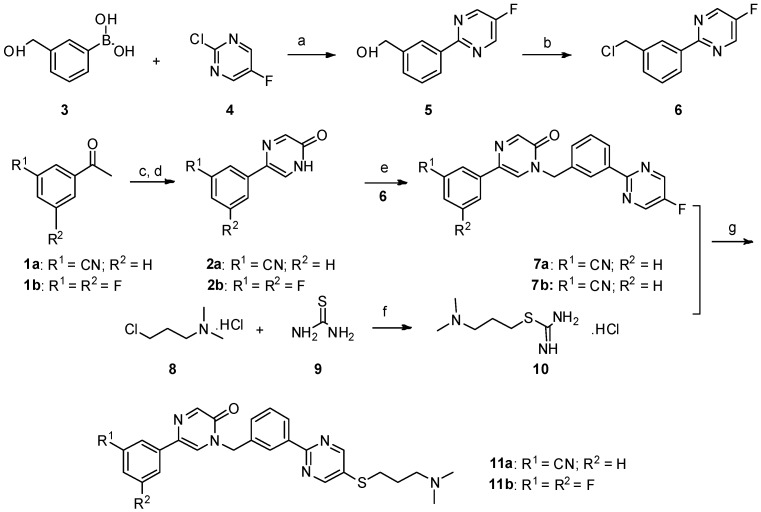
Reagents and conditions: a) PdCl_2_(PPh_3_)_2_, Na_2_CO_3_, ethanol/toluene/water, 90 °C, 8 h; b) SOCl_2_, CHCl_3_, reflux; c) SeO_2_, dioxane:H_2_O = 10:1, reflux, 12 h; d) NaOH, −30 °C; e) NaH, DMF/THF, 0 °C—room temperature, 12 h; f) dry ethanol, reflux; g) NaOH, DMF/H_2_O, 60 °C, 8 h, N_2_.

The synthesis of target compounds **11a** and **11b** is summarized in Scheme 1. The conventional Suzuki coupling reaction of (3-(hydroxymethyl)phenyl)boronic acid and 2-chloro-5-fluoropyrimidine catalyzed with PdCl_2_(PPh_3_)_2_ gave (3-(5-fluoropyrimidin-2-yl)phenyl)methanol **5** with a yield of 68%. Chlorination of **5** in the presence of thionyl chloride provided 2-(3-(chloromethyl)phenyl)-5-fluoropyrimidine **6**. On the other hand, the oxidation of ketones **1a** or **1b** followed by cyclization provided **2a** and **2b** [17], which reacted with compound **6** to give **7a** and **7b**. Compound **10** was synthesized by the reaction of **8** and **9**. Finally, compounds **11a** and **11b** were prepared by the reaction of **7a**, **7b**, and **10** under alkaline conditions.

**Scheme 2:**
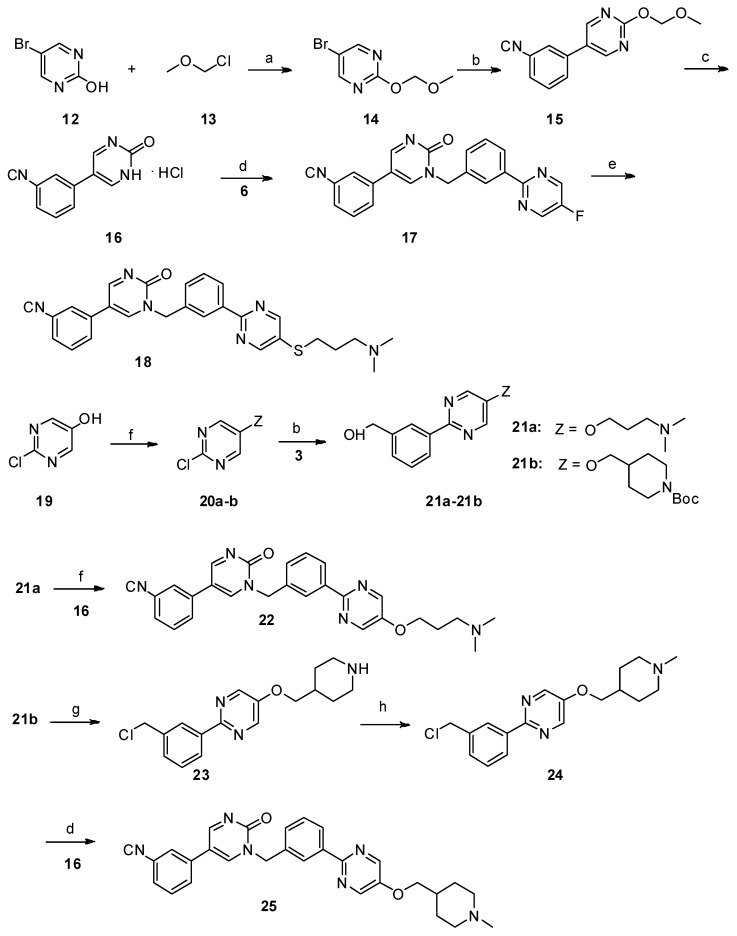
Reagents and conditions: a) *N*,*N*-diisopropylethylamine, dry CH_2_Cl_2_, 0 °C—room temperature, 6 h; b) PdCl_2_(PPh_3_)_2_, Na_2_CO_3_, ethanol/toluene/water, 90 °C, 8 h; c) 10% aq. HCl, MeOH, reflux; d) K_2_CO_3_, dry DMF, 80 °C, 12 h; e) NaOH, DMF/H_2_O, 60 °C, 8 h, N_2_; f) PPh_3_, DIAD, THF, 0 °C—room temperature; g) SOCl_2_, CHCl_3_, reflux; h) 35% formaldehyde, NaBH_4_, MeOH.

The synthesis of compounds **18**, **22**, and **25** is shown in Scheme 2. CH_3_OCH_2_-protected 5-bromopyrimidin-2-ol **14** reacted with (3-cyanophenyl)boronic acid to provide **15**, which was converted to **16** in the presence of 10% hydrogen chloride. Compound **16** reacted with intermediate **6** to provide 3-(1-(3-(5-fluoropyrimidin-2-yl)benzyl)-2-oxo-1. 2-dihydropyrimidin-5-yl)benzonitrile **17**, which reacted with **10** to give target compound **18**. Compound **22** was synthesized from **19** via the Mitsunobu reaction and Suzuki coupling reaction. The reductive amination of **23** with formaldehyde and sodium borohydride gave compound **24**, which reacted with compound **16** under alkaline conditions to provide compound **25**.

**Scheme 3:**
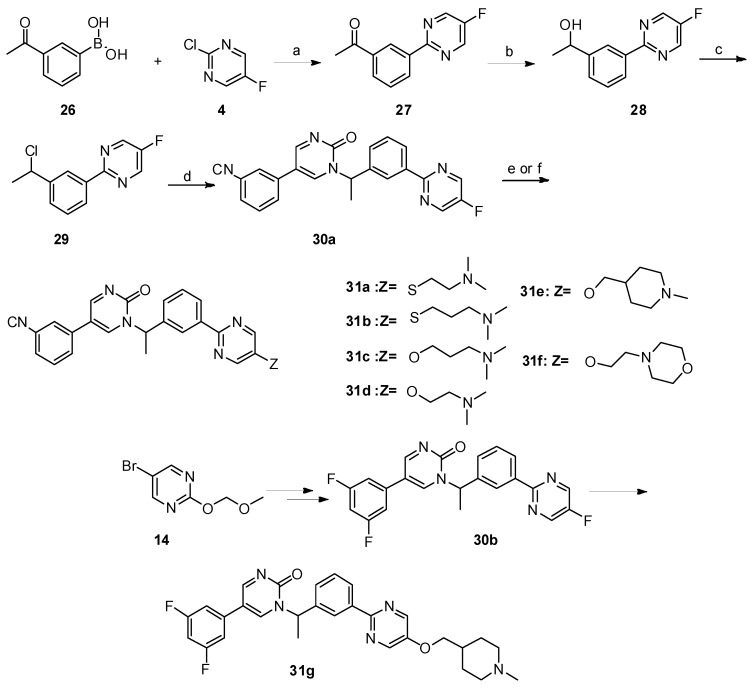
Reagents and conditions: a) PdCl_2_(PPh_3_)_2_, Na_2_CO_3_, ethanol/toluene/water, 90 °C, 8 h; b) NaBH_4_, MeOH, 0 °C—room temperature, 1 h; c) SOCl_2_, CHCl_3_, reflux; d) K_2_CO_3_, dry DMF, 80 °C, 12 h; e) **31a**–**31b**: NaOH, DMF/H_2_O, 60 °C, 8 h, N_2_; f) **31c**–**31g**: NaH, dry DMF, 0 °C—room temperature, 5 h.

The synthesis of compounds **31a**–**g** is summarized in Scheme 3. Intermediate **27** was prepared by the Suzuki coupling reaction of (3-acetylphenyl)boronic acid and 2-chloro-5-fluoropyrimidine. The reduction of compound **27** with sodium borohydride and then chlorination with thionyl chloride produced **29**. In the presence of potassium carbonate, **29** reacted with compound **16** to give **30a**, which reacted with the corresponding amino alcohols or amino thiols to produce target compounds **31a**–**f** (the R and S configurations of **31e** were prepared and are shown in the supporting information). The fluorine-containing target compound **31g** was prepared with a similar procedure, starting from compound **14** and intermediate **30b**.

**Scheme 4:**
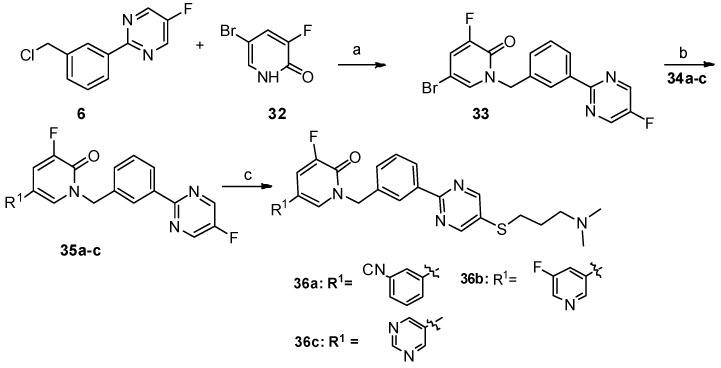
Reagents and conditions: a) K_2_CO_3_, dry DMF, 80 °C, 12 h; b) PdCl_2_(PPh_3_)_2_, Na_2_CO_3_, DME/DMF/water, 89 °C, 12 h; c) NaOH, DMF/H_2_O, 60 °C, 8 h, N_2__._

The synthesis of target compounds **36a**–**c** is listed in Scheme 4. Compound **33** was obtained by the reaction of **6** and commercially available 5-bromo-3-fluoropyridin-2(1*H*)-one **32**. Then, the reaction with aryl boric acids **34a**–**c** afforded the key intermediates, **35a**–**c**, which reacted with **10** in the presence of NaOH to provide target compounds **36a**–**c**.

**Scheme 5:**
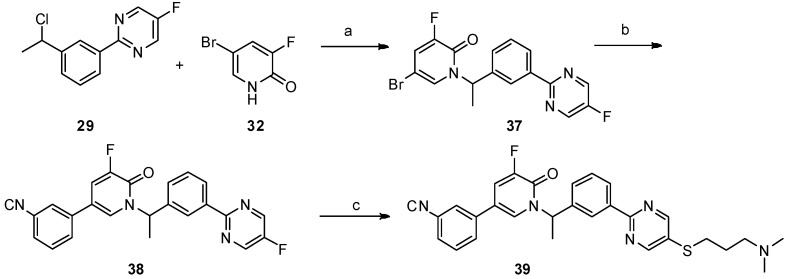
Reagents and conditions: a) K_2_CO_3_, dry DMF, 80 °C, 12 h; b) PdCl_2_(PPh_3_)_2_, Na_2_CO_3_, DME/DMF/water, 89 °C, 12 h; c) NaOH, DMF/H_2_O, 60 °C, 8 h, N_2._

The synthesis of target compound **39** is outlined in Scheme 5. Intermediate **29** reacted with **32** to produce compound **37**. Compound **38** was prepared by the Suzuki coupling reaction of **37** and (3-cyanophenyl)boronic acid. Finally, the reaction of compound **38** with **10** afforded target compound **39**.

### 2.2. Biological Evaluation

#### 2.2.1. In Vitro Growth Inhibition of Human Cancer Cell Lines.

Hepatocellular carcinoma (HCC), a primary liver cancer with a high mortality rate, accounts for 90% of all liver cancers. It is the most common malignant tumor worldwide, especially in Asia, Africa, and southern Europe. China is the “hardest hit” by liver cancer, and the number of annual deaths from this condition in China accounts for about 50% of the total number of liver cancer deaths worldwide. C-Met overexpression, mutational activation, and amplification have been found in some types of cancer, including liver cancer [4,5]. Therefore, hepatic carcinoma 97H cells were selected for the evaluation of the anti-tumor activity of the synthesized compounds, and the results are summarized in Table 1. Most compounds displayed good antiproliferative activity. Among them, compound **31e**, 5-(3-cyanophenyl)-1-(1-(3-(5-((1-methylpiperidin-4-yl)methoxy)pyrimidin-2-yl)phenyl)ethyl)pyrimidin-2(1*H*)-one, exhibited the best result with 26 nM of the IC_50_ value. Further study of the structure–activity relationship indicated the pyridazinone ring or pyrimidin-2(1*H*)-one ring plays an important role in antiproliferative activity. Compounds **11a** and **11b**, pyrazin-2(1*H*)-one derivatives, that were obtained by shifting the nitrogen from the C-1 to C-5 position of pyridazin-3(2*H*)-one, displayed poor activity (IC_50_ values: 9.8 µM for **11a** and 9.1 µM for **11b**). Interestingly, compounds **18** and **25**, which were obtained by shifting the nitrogen from the C-1 to the C-4 position, resulted in significant improvement in anti-proliferative potency (IC_50_: 0.36–0.55 µM). Compounds **31a**–**g**, which have a methyl substitution on the methylene carbon between pyrimidin-2(1*H*)-one and the benzene ring, provided 0.3606 µM to 0.026 µM of the IC_50_ value. This indicates that the methyl substitution at this position is favorable for antiproliferative activity. Compounds **Rac-31e** and **(*R*)**-**31e** with cyclic piperidinylmethyloxy substitution showed excellent activity (IC_50_ = 0.026 µM, 0.018 µM). However, the **(*S*)-31e** almost lost activity (IC_50_ = 1.834 µM). The replacement of the pyridazinone moiety with 3-fluoropyridin-2(1*H*)-one moiety afforded compounds **36a**–**c**. Compound **36a** showed the best result of the 3-fluoropyridin-2(1*H*)-one derivatives, indicating that the benzonitrile group is more favorable than the other two substitutions.

#### 2.2.2. Effect of Compound **31e** on Cell Cycle Progression

**Figure 3:**
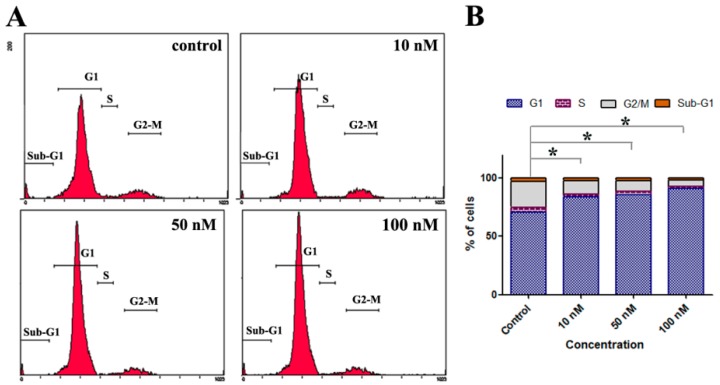
Effect of **31e** on cell cycle progression. (A) 97H cells (3 × 10^5^ cells/sample) were treated with increasing concentrations of **31e** for 24 h, cells were harvested, fixed with 70% ethanol, and stained with propidium iodide (PI). The cellular DNA content was then determined by flow cytometry analysis. (B) Quantitative analysis of the percentage of cells in each cell cycle phase was analyzed by EXPO32 ADC analysis software. The data were presented as the mean ± SEM *P < 0.05, significantly different compared with the control by t test.

To evaluate the ability of compound **31e** to disrupt the regulated cell cycle distribution, we performed flow cytometry analysis to determine the arrest effect of **31e** on the G1 transition, a rigorously regulated process in the cell cycle. The results in Figure 3 show that **31e** can arrest cells in the G1 phase in a dose-dependent manner. When hepatic carcinoma 97H cells were treated with 10 nM of **31e** for 24 h, the population of cells in the G1 phase (83.84%) increased compared to that of the vehicle (DMSO, 70.42%), along with concomitant losses in the G2/M phase. This phenomenon was more obvious at a concentration of 100 nM. A population of up to 90.88% cells was found in the G1 phase. Correspondingly, a population of only 5.78% cells was found in the G2/M phase.

#### 2.2.3. Inhibition of the Migration of Hepatic Carcinoma 97H Cells with Compound **31e**

**Figure 4:**
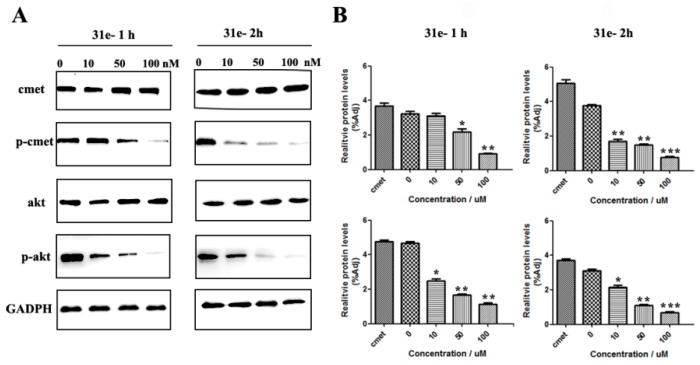
Effect of **31e** on the migration of hepatic carcinoma 97H cells. Hepatic carcinoma 97H cells (50 × 10^4^ cells) suspended in free serum Dulbecco’s Modified Eagle Medium (DMEM) containing **31e** for 36 h were photographed under a phase contrast microscopy (magnification: 4 × objective). All results were expressed as the mean ± SD of at least three independent experiments.

Tumor cell migration is one of the major causes of death in cancer patients. To evaluate the inhibitory ability of **31e** against tumor cell migration, hepatic carcinoma 97H cells (5 × 10^4^ cells per well) were seeded in six-well plates with or without the presence of **31e** and cultured as confluent monolayers. The results shown in Figure 4 demonstrate that **31e** can inhibit tumor cells’ migration. At a concentration of 10 nM, **31e** displayed moderate inhibition of migration. Migration was significantly suppressed when **31e** was used at a dosage of 50 nM. Tumor cells’ migration was completely suppressed with 100 nM of **31e**.

#### 2.2.4. Inhibition of Phosphorylation of c-Met and its Downstream Signaling Component, Akt, with Compound **31e**

**Figure 5:**
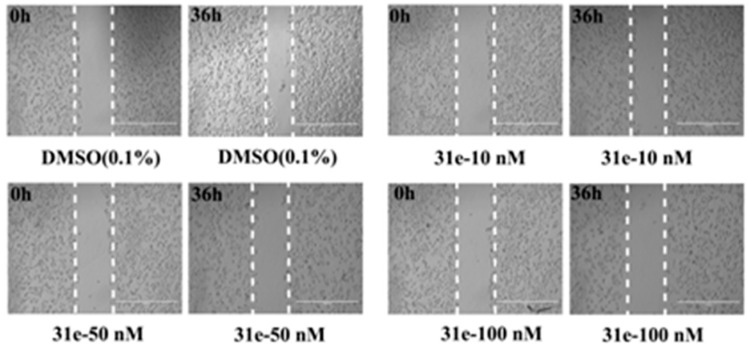
Compound **31e** inhibits c-Met phosphorylation and downstream signaling in cancer cell lines. Cells treated with compound **31e** for 1 h or 2 h were then lysed and subjected to Western blot analysis.

To further study the antiproliferative mechanism of compound **31e**, we analyzed its effects on the phosphorylation of c-Met and its downstream signaling component, Akt, in hepatic carcinoma 97H cells, which overexpress c-Met. As shown in Figure 5A, when hepatic carcinoma 97H cells were treated with compound **31e** at a concentration of 10 or 50 nM for 1 h, the phosphorylation level of Akt and c-Met was suppressed in a dose-dependent manner. When 100 nM of compound **31e** was used, the phosphorylation of both c-Met and Akt was completely inhibited. When 97H cells were treated at the same concentration of compound **31e** for 2 h, the inhibition of c-Met and Akt phosphorylation was more obvious (Figure 5B).

#### 2.2.5. Selectivity of **31e** towards Other Cancer Cells and Normal Human Cells

To evaluate the selectivity of **31e** towards other cancer cells and normal human cells, the antiproliferative activities against Pc9 (human non-small cell lung cancer cells), Hela (human epithelial cervical cancer cells), SJSA1 (human osteosarcoma cells), LO2 (human normal cells), and HLF (human embryonic lung fibroblast cells) were examined. As shown in Table 2, compound **31e** barely inhibited the cancer cells (Pc9, Hela, SJSA1) and normal cells (LO2, HLF), demonstrating that it exclusively inhibits 97H cancer cells. The reason for this remains to be further investigated.

## 3. Experimental Section

### 3.1. Chemistry

^1^H-NMR and ^13^C-NMR spectra were recorded on a Bruker AVANCE 400 or 500 spectrometer (Karlsruhe, Germany). Chemical shifts of protons are reported in parts per million downfield from tetramethylsilane. Peaks are labeled as single (s), broad singlet (br), doublet (d), triplet (t), double doublet (dd), doublet of triplets (dt), or multiplet (m). The high-resolution mass spectra were analyzed on a SHIMADZU LCMS-IT-TOF mass spectrometer. The purity of the synthesized compounds was determined by high-performance liquid chromatography (HPLC) (Agilent, Palo Alto, CA, USA) with a TC-C18 column (250 mm × 4.6 mm, 5 μm) using methanol (1/1000 diethylamine)/water mobile phase (0.50 mL/min). Melting points were determined in open capillary tubes on a MPA100 Optimelt automated melting point system (Stanford Research Systems, San Francisco, CA, USA,). All chemicals were purchased from Sigma-Aldrich and Alfa Aesar chemical companies (Shanghai, China) and were used without further purification.

**Synthesis of 5-(3-cyanophenyl)-1-(3-(5-((3-(dimethylamino)propyl)thio)pyrimidin-2-yl)benzyl)pyrazin-2(1*H*)-one (11a).** In a 200 mL two-necked round-bottomed flask provided with a magnetic stirrer and condenser, 50 mmol of thiourea **9** were dissolved in 60 mL of absolute ethanol. Compound **8** (3.161 mg, 20 mmol) was added in one portion in the thiourea solution. After heating at reflux for 12 h, the reaction mixture was concentrated by rotary evaporation under reduced pressure. The desired compound **10** was obtained as a white solid and was further used without purification [18].

NaOH (135 mg, 3.375 mmol) in 0.5 mL water was added to a mixture of **7a** (287.25 mg, 0.75 mmol) and **10** (444 mg) in DMF (5 mL) under nitrogen, and the mixture was stirred at room temperature for 15 min. Then, the mixture was stirred at 60 °C under nitrogen for 8 h. The reaction mixture was allowed to cool to room temperature. The aqueous phase was extracted with dichloromethane (30 mL × 3). The combined organic layer was washed with H_2_O (15 mL) and brine (10 mL), and then dried over anhydrous Na_2_SO_4_, filtered, and evaporated in vacuo. The residue was purified by flash chromatography over silica gel (DCM/MeOH = 40:1–10:1) to give **5-(3-cyanophenyl)-1-(3-(5-((3-(dimethylamino)propyl)thio)pyrimidin-2-yl)benzyl)pyrazin-2(1*H*)-one (11a)** (224 mg, 62%) as a white solid. m.p. 106.3–107.9 °C. ^1^H-NMR (400 MHz, CDCl_3_) *δ* 8.74 (s, 1H), 8.46–8.39 (m, 2H), 8.32 (s, 1H), 8.01 (s, 1H), 7.93 (d, *J* = 8.0 Hz, 1H), 7.64 (s, 1H), 7.58 (d, *J* = 7.7 Hz, 1H), 7.55–7.47 (m, 3H), 5.27 (s, 2H), 3.03 (t, *J* = 7.2 Hz, 2H), 2.43 (t, *J* = 7.0 Hz, 2H), 2.24 (s, 6H), 1.91–1.76 (m, 2H); ^13^C-NMR (101 MHz, CDCl_3_) *δ* 161.18, 157.41, 155.35, 149.17, 138.08, 136.67, 134.95, 131.70, 131.28, 130.82, 129.79, 129.70, 129.08, 128.52, 128.04, 125.10, 118.62, 113.06, 57.87, 52.29, 45.34, 31.60, 27.04. HRMS (ESI) calculated for C_27_H_26_N_6_OS [M + H]^+^: 483.1962, found: 483.1947. Purity: 98.3% (by HPLC).

**5-(3,5-difluorophenyl)-1-(3-(5-((3-(dimethylamino)propyl)thio)pyrimidin-2-yl)benzyl)pyrazin-2(1*H*)-one (11b)**. Compound **11b** was prepared via a similar procedure of **11a**. White solid. m.p. 143.7–145.2 °C. Yield: 65%. ^1^H-NMR (500 MHz, CDCl_3_) *δ* 8.74(s, 2H), 8.46–8.39 (m, 2H), 8.30 (s, 1H), 7.56 (s, 1H), 7.54–7.46 (m, 2H), 7.25–7.18 (m, 2H), 6.82–6.65 (m, 1H), 5.25 (s, 2H), 3.03 (t, *J* = 7.2 Hz, 2H), 2.42 (t, *J* = 7.0 Hz, 2H), 2.24 (s, 6H), 1.92–1.76 (m, 2H). ^13^C-NMR (126 MHz, CDCl_3_) *δ* 164.52 (*J_CF_* = 247.5 Hz), 163.42 (*J_CF_* = 246.25 Hz), 161.21, 157.45, 155.37, 148.97, 138.74 (*J_CF_* = 10.0 Hz), 138.10, 134.92, 131.64 (*J*_CF_ = 3.0 Hz), 130.81, 130.76, 129.76, 128.49, 128.00, 125.03, 107.79 (*J_CF_* = 26.25 Hz), 107.78 (*J_CF_* = 13.75 Hz), 103.21 (*J_CF_* = 26.25 Hz), 57.89, 52.24, 45.38, 31.65, 27.11. HRMS (ESI) calculated for C_26_H_25_N_5_OF_2_S [M + H]^+^: 494.1821, found: 494.1823. Purity: 99.7% (by HPLC).

**5-(3-cyanophenyl)-1-(3-(5-((3-(dimethylamino)propyl)thio)pyrimidin-2-yl)benzyl)pyrimidin-2(1*H*)-one (18)**. Compound **18** was prepared via a similar procedure of **11a**. White oil. Yield: 77.1%. ^1^H-NMR (500 MHz, CDCl_3_) *δ* 8.82 (d, *J* = 3.2 Hz, 1H), 8.69 (s, 2H), 8.42 (s, 1H), 8.38 (d, *J* = 7.6 Hz, 1H), 7.99 (d, *J* = 3.2 Hz, 1H), 7.65 (s, 1H), 7.60 (t, *J* = 7.1 Hz, 2H), 7.55–7.46 (m, 3H), 5.28 (s, 2H), 3.01 (t, *J* = 7.2 Hz, 2H), 2.40 (t, *J* = 7.0 Hz, 2H), 2.21 (s, 6H), 1.86–1.76 (m, 2H). ^13^C-NMR (126 MHz, CDCl_3_) *δ* 164.70, 161.23, 157.43, 155.73, 145.11, 138.15, 135.10, 134.64, 131.48, 131.11, 130.93, 130.32, 130.14, 129.83, 129.37, 128.58, 128.18, 118.25, 116.63, 57.95, 54.44, 45.43, 31.67, 27.17. HRMS (ESI) calculated for C_27_H_26_N_6_OS [M + H]^+^: 483.1962, found: 483.1942. Purity: 98.3% (by HPLC).

**Synthesis of 5-(3-cyanophenyl)-1-(3-(5-(3-(dimethylamino)propoxy)pyrimidin-2-yl)benzyl)pyrimidin-2(1*H*)-one (22)**. Compound **21a** (157.85 mg, 0.55 mmol) and triphenylphosphine (328 mg, 1.25 mmol) were added successively to a suspension of **16** (116.75 mg, 0.5 mmol) in THF (5 mL) under nitrogen. A solution of diisopropyl azodicarboxylate (104.49 mg, 0.6 mmol) in THF (1 mL) was then slowly added dropwise with ice cooling. The resultant solution was stirred at room temperature for 12 h. The aqueous phase was extracted with dichloromethane (30 mL × 3). The combined organic layer was washed with H_2_O (20 mL) and brine (10 mL), and then dried over anhydrous Na_2_SO_4_, filtered, and evaporated in vacuo. The residue was purified by flash chromatography over silica gel (DCM/MeOH = 40:1–10:1) to give **22** [14]. Yellow solid. m.p. 122.3–123.6 °C. Yield: 45%. ^1^H-NMR (500 MHz, CDCl_3_) *δ* 8.84 (d, *J* = 3.0 Hz, 1H), 8.45 (s, 2H), 8.40–8.33 (m, 2H), 7.91 (d, *J* = 3.0 Hz, 1H), 7.66 –7.57 (m, 3H), 7.56–7.47 (m, 3H), 5.29 (s, 2H), 4.18 (t, *J* = 6.2 Hz, 2H), 2.53 (t, *J* = 6.2 Hz, 2H), 2.31 (s, 6H), 2.07–2.00 (m, 2H). ^13^C-NMR (126 MHz, CDCl_3_) *δ* 164.56, 156.67, 155.65, 151.81, 144.80, 143.87, 138.53, 134.73, 134.60, 131.43, 130.25, 130.20, 130.09, 129.76, 129.26, 128.14, 127.78, 118.14, 116.54, 113.67, 67.04, 55.84, 54.31, 45.37, 27.17. HRMS (ESI) calculated for C_27_H_26_N_6_O_2_ [M + H]^+^: 467.2190, found: 467.2185. Purity: 96.784% (by HPLC).

**Synthesis of 5-(3-cyanophenyl)-1-(3-(5-((1-methylpiperidin-4-yl)methoxy)pyrimidin-2-yl)benzyl)pyrimidin-2(1*H*)-one (25).** 2-(3-(chloromethyl)phenyl)-5-((1-methylpiperidin-4-yl)methoxy)pyrimidine **24** (99 mg, 0.3 mmol, 1 eq.) and potassium carbonate (124 mg, 0.9 mmol, 3 eq.) were added to a suspension of **16** (77 mg, 0.33 mmol) in dry DMF (2 mL), and the mixture was stirred at 80 °C for 12 h. The reaction mixture was allowed to cool to room temperature. The aqueous phase was extracted with dichloromethane (10 mL × 3). The combined organic layer was washed with H_2_O (5 mL) and brine (10 mL), and then dried over anhydrous Na_2_SO_4_, filtered, and evaporated in vacuo. The residue was purified by flash chromatography over silica gel (DCM/MeOH = 30:1–10:1) to give compound **25** [15]. White solid. m.p. 116.7–118.2 °C. Yield: 48%. ^1^H-NMR (500 MHz, CDCl_3_) *δ* 8.84 (d, *J* = 2.9 Hz, 1H), 8.44 (s, 2H), 8.39–8.33 (m, 2H), 7.89 (d, *J* = 2.6 Hz, 1H), 7.65–7.60 (m, 2H), 7.60–7.56 (m, 1H), 7.56–7.47 (m, 3H), 5.29 (s, 2H), 3.96 (d, *J* = 5.6 Hz, 2H), 2.98 (d, *J* = 11.1 Hz, 2H), 2.34 (s, 3H), 2.04 (t, *J* = 11.8 Hz, 2H), 1.89–1.81 (m, 3H), 1.54–1.46 (m, 2H). ^13^C-NMR (126 MHz, CDCl_3_) *δ* 164.56, 156.70, 155.63, 151.85, 144.72, 143.82, 138.52, 134.73, 134.61, 131.43, 130.25, 130.22, 130.08, 129.77, 129.26, 128.15, 127.77, 118.13, 116.53, 113.68, 73.28, 55.16, 54.30, 46.21, 35.17, 28.69. HRMS (ESI) calculated for C_29_H_28_N_6_O_2_ [M + H]^+^: 493.2347, found: 493.2351. Purity: 97.8% (by HPLC).

**5-(3-cyanophenyl)-1-(1-(3-(5-((2-(dimethylamino)ethyl)thio)pyrimidin-2-yl)phenyl)ethyl)pyrimidin-2(1*H*)-one (31a).** Compound **31a** was prepared via a similar procedure of **11a**. Yellow oil. Yield: 66%. **^1^H NMR** (400 MHz, CDCl_3_) *δ* 8.78 (d, *J* = 3.3 Hz, 1H), 8.74 (s, 2H), 8.48 (s, 1H), 8.42 (d, *J* = 7.5 Hz, 1H), 7.71 (d, *J* = 3.3 Hz, 1H), 7.63–7.53 (m, 3H), 7.52–7.45 (m, 3H), 6.35 (q, *J* = 6.9 Hz, 1H), 3.08 (t, *J* = 7.1 Hz, 2H), 2.60 (t, *J* = 7.1 Hz, 2H), 2.28 (s, 6H), 1.90 (d, *J* = 7.0 Hz, 3H). ^13^C-NMR (126 MHz, CDCl_3_) *δ* 164.01, 161.33, 157.54, 155.44, 142.40, 138.86, 138.26, 134.89, 131.45, 130.97, 130.31, 130.15, 129.84, 129.32, 128.64, 126.92, 118.22, 116.76, 113.72, 58.36, 56.32, 45.37, 32.02, 19.19. HRMS (ESI) calculated for C_27_H_26_N_6_OS [M + H]^+^: 483.1962, found: 483.1949. Purity: 98% (by HPLC).

**5-(3-cyanophenyl)-1-(1-(3-(5-((3-(dimethylamino)propyl)thio)pyrimidin-2-yl)phenyl)ethyl)pyrimidin-2(1*H*)-one (31b).** Compound **31b** was prepared via a similar procedure of **11a**. Yellow oil. Yield: 75%. **^1^H NMR** (500 MHz, CDCl_3_) *δ* 8.78 (d, *J* = 3.2 Hz, 1H), 8.73 (s, 2H), 8.49 (s, 1H), 8.42 (d, *J* = 7.6 Hz, 1H), 7.70 (d, *J* = 3.2 Hz, 1H), 7.63–7.58 (m, 1H), 7.57–7.46 (m, 5H), 6.36 (q, *J* = 6.9 Hz, 1H), 3.03 (t, *J* = 7.2 Hz, 2H), 2.42 (t, *J* = 7.0 Hz, 3H), 2.23 (s, 6H), 1.91 (d, *J* = 7.0 Hz, 3H), 1.88–1.77 (m, 2H). ^13^C-NMR (126 MHz, CDCl_3_) *δ* 164.04, 161.27, 157.50, 155.49, 142.43, 138.82, 138.32, 134.88, 131.49, 131.02, 130.33, 130.31, 130.18, 129.86, 129.34, 128.66, 126.92, 118.24, 116.83, 113.74, 57.97, 56.37, 45.46, 31.70, 27.18, 19.21. HRMS (ESI) calculated for C_28_H_28_N_6_OS [M + H]^+^: 497.2118, found: 497.2108. Purity: 97.1% (by HPLC).

**Synthesis of 5-(3-cyanophenyl)-1-(1-(3-(5-(3-(dimethylamino)propoxy)pyrimidin-2-yl)phenyl)ethyl)pyrimidin-2(1*H*)-one (31c).** A solution of 3-(dimethylamino)propan-1-ol (113.3 mg, 1.1 mmol, 2 eq.) in dry DMF( 5 mL) was added to a suspension of NaH (43.56 mg, 3.3 eq.) in dry DMF (5 mL) at 0 °C. After 30 mins, 5-(3-cyanophenyl)-1-(1-(3-(5-fluoropyrimidin-2-yl)phenyl)ethyl)pyrimidin-2(1*H*)-one **30a** (210 mg, 0.55 mmol, 1 eq.) was added. After the reaction mixture was stirred for 5 h, the reaction was quenched with water (15 mL) and the mixture was extracted with dichloromethane (20 mL × 3). The combined organic layer was dried over anhydrous Na_2_SO_4_ and filtered. After the solvent was removed in vacuo, the crude product was purified by column chromatography (DCM/MeOH = 50:1–10:1) to afford **31c** as a white solid [19]. m.p. 68.3–69.9 °C. Yield: 62%. ^1^H-NMR (400 MHz, CDCl_3_) *δ* 8.78 (d, *J* = 3.4 Hz, 1H), 8.46 (s, 2H), 8.42 (s, 1H), 8.35 (d, *J* = 7.7 Hz, 1H), 7.73 (d, *J* = 3.4 Hz, 1H), 7.64–7.58 (m, 1H), 7.56–7.42 (m, 5H), 6.35 (q, *J* = 7.0 Hz, 1H), 4.21 (t, *J* = 6.1 Hz, 2H), 2.75 (t, *J* = 7.3 Hz, 2H), 2.47 (s, 6H), 2.24–2.09 (p, 2H), 1.90 (d, *J* = 7.0 Hz, 3H). ^13^C-NMR (101 MHz, CDCl_3_) *δ* 163.98, 156.99, 155.48, 151.75, 144.00, 142.53, 138.71, 138.62, 134.92, 131.44, 130.32, 130.19, 129.77, 129.44, 129.35, 128.22, 126.55, 118.28, 116.76, 113.69, 66.75, 56.44, 55.79, 44.79, 26.49, 19.24. HRMS (ESI) calculated for C_28_H_28_N_6_O_2_ [M + HCOO]^−^: 525.2256, found: 525.2238. Purity: 98.3% (by HPLC).

**5-(3-cyanophenyl)-1-(1-(3-(5-(2-(dimethylamino)ethoxy)pyrimidin-2-yl)phenyl)ethyl)pyrimidin-2(1*H*)-one (31d).** Compound **31d** was prepared via a similar procedure of **31c**. White solid. m.p. 53.3-55.0 °C. Yield: 74%. ^1^H-NMR (400 MHz, CDCl_3_) *δ* 8.80 (d, *J* = 3.3 Hz, 1H), 8.51 (s, 2H), 8.45 (s, 1H), 8.38 (d, *J* = 7.7 Hz, 1H), 7.72 (d, *J* = 3.4 Hz, 1H), 7.64–7.58 (m, 1H), 7.57–7.42 (m, 5H), 6.38 (q, *J* = 7.0 Hz, 1H), 4.24 (t, *J* = 5.4 Hz, 2H), 2.83 (t, *J* = 5.4 Hz, 2H), 2.40 (s, 6H), 1.91 (d, *J* = 7.0 Hz, 3H). ^13^C-NMR (101 MHz, CDCl_3_) *δ* 163.94, 156.94, 155.45, 151.78, 144.05, 142.52, 138.67, 138.59, 134.89, 131.40, 130.27, 130.15, 129.73, 129.44, 129.32, 128.19, 126.53, 118.24, 116.72, 113.66, 66.88, 58.10, 56.40, 45.90, 19.18. HRMS (ESI) calculated for C_27_H_26_N_6_O_2_ [M + HCOO]^−^: 511.2099, found: 511.2074. Purity: 98% (by HPLC).

**5-(3-cyanophenyl)-1-(1-(3-(5-((1-methylpiperidin-4-yl)methoxy)pyrimidin-2-yl)phenyl)ethyl)pyrimidin-2(1*H*)-one (31e).** Compound **31e** was prepared via a similar procedure of **31c**. Yellow solid. m.p. 98.2–99.9 °C. Yield: 76%. ^1^H-NMR (500 MHz, DMSO-*d*_6_) *δ* 9.04 (d, *J* = 3.2 Hz, 1H), 8.72 (d, *J* = 3.2 Hz, 1H), 8.64 (s, 2H), 8.33 (s, 1H), 8.27–8.18 (m, 2H), 8.03 (d, *J* = 8.0 Hz, 1H), 7.81 (d, *J* = 7.7 Hz, 1H), 7.65 (t, *J* = 7.8 Hz, 1H), 7.59–7.45 (m, 2H), 6.02 (q, *J* = 7.0 Hz, 1H), 4.04 (d, *J* = 5.8 Hz, 2H), 2.80 (d, *J* = 11.1 Hz, 2H), 2.16 (s, 3H), 1.92 (d, *J* = 7.2 Hz, 3H), 1.87 (d, *J* = 11.3 Hz, 2H), 1.78–1.67 (m, 3H), 1.36–1.27 (m, 2H). ^13^C-NMR (101 MHz, CDCl_3_) *δ* 163.97, 156.82, 155.49, 151.98, 143.94, 142.52, 138.66, 134.92, 131.44, 130.30, 130.17, 129.77, 129.44, 129.34, 128.21, 126.52, 118.26, 116.75, 113.71, 73.39, 56.42, 55.30, 46.35, 35.26, 28.81, 19.21. HRMS (ESI) calculated for C_30_H_30_N_6_O_2_ [M + HCOO]^−^: 551.2412, found: 551.2402. Purity: 98.9% (by HPLC).

**5-(3-cyanophenyl)-1-(1-(3-(5-(2-morpholinoethoxy)pyrimidin-2-yl)phenyl)ethyl)pyrimidin-2(1*H*)-one (31f).** Compound **31f** was prepared via a similar procedure of **31c**. White solid. m.p. 82.3–83.9 °C. Yield: 68%. ^1^H-NMR (400 MHz, CDCl_3_) *δ* 8.78 (d, *J* = 3.4 Hz, 1H), 8.47 (s, 2H), 8.43 (s, 1H), 8.35 (d, *J* = 7.7 Hz, 1H), 7.72 (d, *J* = 3.4 Hz, 1H), 7.62–7.56 (m, 1H), 7.56–7.42 (m, 5H), 6.35 (q, *J* = 7.0 Hz, 1H), 4.25 (t, *J* = 5.5 Hz, 2H), 3.79–3.66 (m, 4H), 2.85 (t, *J* = 5.5 Hz, 2H), 2.68–2.50 (m, 4H), 1.89 (d, *J* = 7.0 Hz, 3H). ^13^C-NMR (101 MHz, CDCl_3_) *δ* 163.95, 157.05, 155.45, 151.72, 144.13, 142.50, 138.70, 138.56, 134.91, 131.42, 130.28, 130.15, 129.75, 129.46, 129.33, 128.21, 126.56, 118.25, 116.72, 113.68, 66.87, 66.77, 57.53, 56.39, 54.17, 19.21. HRMS (ESI) calculated for C_29_H_28_N_6_O_3_ [M + HCOO]^−^: 553.2205, found: 553.2181. Purity: 99.6% (by HPLC).

**5-(3,5-difluorophenyl)-1-(1-(3-(5-((1-methylpiperidin-4-yl)methoxy)pyrimidin-2-yl)phenyl)ethyl)pyrimidin-2(1*H*)-one (31g).** Compound 31g was prepared via a similar procedure of 31c. White solid. m.p. 86.9–88.6 °C. Yield: 55%. ^1^H-NMR (500 MHz, CDCl_3_) *δ* 8.76 (d, *J* = 3.2 Hz, 1H), 8.43 (s, 2H), 8.42 (s, 2H), 8.35 (d, *J* = 7.7 Hz, 1H), 7.67 (d, *J* = 3.3 Hz, 1H), 7.51 (t, *J* = 7.7 Hz, 1H), 7.47–7.39 (m, 1H), 6.82–6.69 (m, 3H), 6.35 (q, *J* = 6.9 Hz, 1H), 3.95 (d, *J* = 5.7 Hz, 2H), 3.00 (d, *J* = 11.2 Hz, 2H), 2.35 (s, 3H), 2.08 (t, *J* = 11.5 Hz, 2H), 1.93–1.77 (m, 6H), 1.60–1.49 (m, 2H). ^13^C-NMR (126 MHz, CDCl_3_) *δ* 163.92, 163.665(*J_CF_* = 248.75 Hz), 163.565(*J_CF_* = 248.75 Hz), 156.88, 155.54, 151.94, 143.92, 142.42, 138.69, 138.65, 136.67 (*J_CF_* = 10.0 Hz), 129.74, 129.36, 128.18, 126.59, 116.74 (*J_CF_* = 2.2 Hz),108.83 (*J_CF_* = 12.5 Hz), 108.825(*J_CF_* = 26.25 Hz), 103.41 (*J_CF_* = 25.2 Hz), 73.28, 56.39, 55.20, 46.15, 35.17, 28.61, 19.18. HRMS (ESI) calculated for C_29_H_29_N_5_O_2_F_2_ [M + HCOO]^−^: 562.2271, found: 562.2248. Purity: 99.4% (by HPLC).

**5-(3-cyanophenyl)-1-(3-(5-((3-(dimethylamino)propyl)thio)pyrimidin-2-yl)benzyl)-3-fluoropyridin-2(1*H*)-one (36a).** Compound **36a** was prepared via a similar procedure of **11a**. Yellow solid. m.p. 116.3–117.9 °C. Yield: 72%. ^1^H-NMR (400 MHz, CDCl_3_) *δ* 8.74 (s, 1H), 8.43 (s, 1H), 8.41–8.37 (m, 1H), 7.64 (s, 1H), 7.62–7.52 (m, 3H), 7.52–7.49 (m, 2H), 7.45–7.41 (m, 1H), 7.37 (dd, *J* = 9.8, 2.3 Hz, 1H), 5.36 (s, 2H), 3.03 (t, *J* = 7.2 Hz, 2H), 2.45 (t, *J* = 7.0 Hz, 2H), 2.26 (s, 6H), 1.93–1.80 (m, 2H). ^13^C-NMR (126 MHz, CDCl_3_) *δ* 161.35, 157.48, 155.62 (*J_CF_* = 26.25 Hz), 152.58 (*J_CF_* = 251.25 Hz), 137.92, 137.05, 135.88, 131.10, 130.72, 130.68, 130.23, 130.16(*J_CF_* = 3.75 Hz), 130.06, 129.66, 129.42, 128.25, 127.85, 119.54 (*J_CF_* = 17.5 Hz), 118.29, 116.57 (*J_CF_* = 5.0 Hz), 113.46, 57.86, 52.50, 45.31, 31.65, 27.03. HRMS (ESI) calculated for C_28_H_26_N_5_OFS [M + H]^+^: 500.1915, found: 500.1898. Purity: 98.1% (by HPLC).

**1-(3-(5-((3-(dimethylamino)propyl)thio)pyrimidin-2-yl)benzyl)-5,5′-difluoro-[3,3′-bipyridin]-6(1H)-one (36b).** Compound **36b** was prepared via a similar procedure of **11a**. White solid. m.p. 128.3–129.9 °C. Yield: 75%. ^1^H-NMR (400 MHz, CDCl_3_) *δ* 8.74 (s, 2H), 8.47–8.36 (m, 4H), 7.51 (d, *J* = 5.1 Hz, 2H), 7.45–7.42 (m, 1H), 7.40–7.33 (m, 2H), 5.37 (s, 2H), 3.04 (t, *J* = 7.2 Hz, 2H), 2.44 (t, *J* = 7.0 Hz, 2H), 2.25 (s, 6H), 1.91–1.79 (m, 2H). ^13^C-NMR (126 MHz, CDCl_3_) *δ* 161.29, 159.52 (*J_CF_* = 257.50 Hz), 157.52, 157.45, 155.59 (*J_CF_* = 26.25 Hz), 152.66 (*J_CF_* = 252.50 Hz), 142.80 (*J_CF_* = 3.75 Hz), 137.96, 137.20 (*J_CF_* = 23.75Hz), 135.75, 130.77, 130.73, 130.36 (*J_CF_* = 5.0 Hz), 129.65, 128.26, 127.88, 120.01 (*J_CF_* = 18.75 Hz), 119.33 (*J_CF_* = 18.75 Hz), 113.96 (*J_CF_* = 6.25 Hz), 57.91, 52.48, 45.40, 31.69, 27.16. HRMS (ESI) calculated for C_26_H_25_N_5_OF_2_S [M + H]^+^: 494.1821, found: 494.1807. Purity: 99.7% (by HPLC).

**1-(3-(5-((3-(dimethylamino)propyl)thio)pyrimidin-2-yl)benzyl)-3-fluoro-5-(pyrimidin-5-yl)pyridin-2(1H)-one (36c).** Compound **36c** was prepared via a similar procedure of **11a**. White solid. 128.9–129.8 °C. Yield: 78%. ^1^H-NMR (400 MHz, CDCl_3_) *δ* 9.16 (s, 1H), 8.75 (s, 2H), 8.74 (s, 2H), 8.45–8.37 (m, 2H), 7.51 (d, *J* = 5.1 Hz, 2H), 7.44 (s, 1H), 7.36 (dd, *J* = 9.5, 2.3 Hz, 1H), 5.37 (s, 2H), 3.04 (t, *J* = 7.2 Hz, 2H), 2.45 (t, *J* = 7.0 Hz, 2H), 2.26 (s, 6H), 1.93–1.80 (m,2H). ^13^C-NMR (126 MHz, CDCl_3_) *δ* 161.06, 157.52, 157.25, 155.40 (*J_CF_* = 25.7 Hz), 153.68, 151.64, 137.82, 135.46, 131.62 (*J_CF_* = 10.2 Hz), 130.63, 130.16 (*J_CF_* = 4.6 Hz), 129.50, 128.71 (*J_CF_* = 12.5 Hz), 128.14, 127.73, 118.68 (*J_CF_* = 18.2 Hz), 111.65 (*J_CF_* = 5.5 Hz), 57.73, 52.35, 45.21, 31.49, 26.96. HRMS (ESI) calculated for C_25_H_25_N_6_OFS [M + H]^+^: 477.1867, found: 477.1857. Purity: 98.9% (by HPLC).

**5-(3-cyanophenyl)-1-(1-(3-(5-((3-(dimethylamino)propyl)thio)pyrimidin-2-yl)phenyl)ethyl)-3-fluoropyridin-2(1*H*)-one (39).** Compound **39** was prepared via a similar procedure of **11a**. Colorless oil. Yield: 82%. ^1^H-NMR (400 MHz, CDCl_3_) *δ* 8.75 (s, 2H), 8.49 (s, 1H), 8.40 (d, *J* = 7.6 Hz, 1H), 7.60–7.43 (m, 6H), 7.32 (dd, *J* = 9.8, 2.3 Hz, 1H), 7.22 (s, 1H), 6.62 (q, *J* = 7.0 Hz, 1H), 3.04 (t, *J* = 7.2 Hz, 2H), 2.47 (t, *J* = 7.0 Hz, 2H), 2.27 (s, 6H), 1.91–1.81 (m, 5H). ^13^C-NMR (101 MHz, CDCl_3_) *δ* 161.36, 157.45, 155.42(*J_CF_* = 25.0 Hz), 152.15(*J_CF_* = 251.0 Hz), 139.61, 137.97, 137.32, 130.98, 130.76, 130.21, 130.03, 130.01, 129.53, 129.40, 128.20, 127.26 (*J_CF_* = 5.0 Hz), 126.64, 119.00 (*J_CF_* = 18.0 Hz), 118.33, 116.57 (*J_CF_* = 6.0 Hz), 113.37, 57.88, 53.78, 45.34, 31.65, 27.08, 19.23. HRMS (ESI) calculated for C_29_H_28_N_5_OFS [M + H]^+^: 514.2071, found: 514.2066. Purity: 99% (by HPLC).

### 3.2. Biological Assay

#### 3.2.1. Cell lines and Culture

The cell lines, 97H (human liver cancer cell), Pc9 (human non-small cell lung cancer), HELA (human epithelial cervical cancer cell line), SJSA1 (human osteosarcoma cells), LO2 (human normal liver cells), and HLF (human embryonic lung fibroblast), used in this study were purchased from the Guangzhou ginny ou Biotechnology Co. Ltd. (Guangzhou, China). Cell lines were cultivated in Dulbecco’s Modified Eagle Medium (DMEM) containing 10% (*v*/*v*) heat-inactivated fetal bovine serum (FBS), 100 units/mL penicillin, and 100 μg/mL streptomycin.

#### 3.2.2. Kinase Inhibition Assay.

The c-Met enzymes and the Z’-LYTE Kinase Kit were purchased from Invitrogen. The tested compounds were used in concentrations of 0.001 to 10 mM. Briefly, 1 μL of an inhibitor or 5% DMSO, 2 μL of enzyme, and 2 μL of substrate/ATP mix were added to a 384-well low volume plate L and incubated at room temperature for 60 min. Then, 5 μL of ADP-Glo™ Reagent was added, and the mixture was incubated at room temperature for 40 min. Finally, 10 μL of Kinase Detection Reagent was added, and the mixture was incubated at room temperature for 30 min. The plate was measured on a multifunction microplate reader (Molecular Devices, Flex Station 3), and the luminescence was recorded (integration time 0.5–1 s). Curve fitting and data presentations were performed using Graph Pad Prism version 5.0 (GraphPad Inc., La Jolla, CA, USA). Every experiment was repeated at least three times.

#### 3.2.3. MTT (Thiazolyl Blue Tetrazolium Bromide) Assay.

For the cytotoxicity assay, the cells grown in the logarithmic phase were seeded into 96-well plates (5 × 10^3^ cells/well) for 24 h, and then exposed to different concentrations of the test compounds for 48 h. After the attached cells had been incubated with 5 mg/mL MTT (Sigma, St. Louis, MO, USA) for another 4 h, the suspension was discarded and subsequently the dark blue crystals (formazan) were solubilized in dimethyl sulfoxide (DMSO). Then, the absorbance was measured at 570 nm using a multifunction microplate reader (Molecular Devices, Flex Station 3) (Thermo Fisher Scientific, San Jose, CA, USA), and each experiment was performed at least in triplicate. The cytotoxic effects of each compound were expressed as the IC_50_ values, which represents the drug concentration required to cause 50% tumor cell growth inhibition. This was calculated with GraphPad Prism Software version 5.02 (GraphPad Inc., La Jolla, CA, USA).

#### 3.2.4. Western Blot Analysis.

97H cells seeded in 60-mm dishes at a density of 5 × 10^5^ cells/well were incubated with or without compound **31e** at the indicated concentrations for 4 h. After incubation, the cells were washed twice with ice-cold PBS, and then lysed in RIPA lysis buffer (Radio Immunoprecipitation Assay) containing 150 mM NaCl, 50 mM Tris (pH 7.4), 1% (*w*/*v*) sodium deoxycholate, 1% (*v*/*v*) Triton X-100, 0.1% (*w*/*v*) SDS, and 1 mM EDTA (ethylenediaminetetraacetic acid) (Beyotime, Shanghai, China). The lysates were incubated at 0 °C for 30 min, and vortexed every 10 min intermittently, then the total proteins were harvested by centrifuging at 12,500 g for 15 min. The protein concentrations were determined by a BCA (bicinchoninic acid) Protein Assay Kit (Thermo Fisher Scientific, Rockford, IL, USA), and then the protein extracts were reconstituted in loading buffer containing 62 mM Tris-HCl, 2% SDS (sodium dodecyl sulfate), 10% glycerol, and 5% β-mercaptoethanol (Beyotime, Shanghai, China), and boiled at 100 °C for 3 min. An equal amount of the proteins (40 μg) was separated by 8%–12% sodium dodecyl sulphate-polyacrylamide gel electrophoresis (SDS-PAGE) and were transferred to nitrocellulose membranes (Amersham Biosciences, Little Chalfont, Buckinghamshire, UK). After blocking with 5% non-fat dried milk in TBS containing 1% Tween-20 for 90 min at room temperature, the membranes were incubated overnight with specific primary antibodies (Abcam, London, England) at 4 °C. After three washes in TBST, the membranes were incubated with the appropriate HRP-conjugated (Horseradish Peroxidase-conjugated) secondary antibodies at room temperature for 2 h. The blots were developed with enhanced chemiluminescence (Pierce, Rockford, IL, USA) and detected by an LAS4000 imager (GE Healthcare, Waukesha, WI, USA).

#### 3.2.5. Cell Cycle Analysis

97H cells were seeded in 6-well plates (3 × 10^5^ cells/well) and incubated in the presence or absence of compound **31e** at the indicated concentrations for 24 h. Then, cells were harvested by centrifugation and fixed in 70% ice-cold ethanol overnight. The ethanol was removed the next day, and then the cells were resuspended in the ice-cold PBS and treated with RNAse A (Keygen Biotech, Nanjing, China) at 37 °C for 30 min, followed by incubation with the DNA staining solution propidium iodide (PI) (Keygen Biotech, Nanjing, China) at 4 °C for 30 min. About 10,000 events were detected by flow cytometry (Epics XL, Beckman Coulter, Fullerton, CA, USA) at 488 nm. The data regarding the number of cells in different phases of the cell cycle were analyzed by EXPO32 ADC analysis software (Beckman Coulter, Fullerton, CA, USA).

#### 3.2.6. Anti-Cell-Migration Study

97H cells were plated in a 6-well culture dish at 5 × 10^4^ cells/dish and grown for 24 h, and the non-migrated cells were scraped off the upper surface of the membrane with a 10 μL pipette. The medium was then replaced with 10% serum DMEM medium and treated with compound **31e** at the indicated concentrations for another 36 h. After washing with phosphate buffer solution (PBS), the cell images were immediately detected by a Zeiss LSM 570 laser scanning confocal microscope (Carl Zeiss, Jena, Germany).

## 4. Conclusions

We synthesized a series of new Tepotinib derivatives to evaluate their ability to act as antiproliferative agents. The optimal compound, **31e**, in which pyridazinone is replaced with the pyrimidin-2(1*H*)-one moiety and methyl substitution on the methylene moiety of Tepotinib is introduced, exhibited potent antiproliferative activity (IC_50_: 0.026 μΜ) against hepatic carcinoma 97H cells. The mechanism study indicated that **31e** induces G1 phase arrest and inhibits the phosphorylation of c-Met and its downstream signaling component, Akt, in a dose-dependent manner. Overall, the current study demonstrates that **31e** is a promising leading compound in the development of anticancer agents. Further structural optimization and the evaluation of its in vivo anti-tumor activity are in progress.

## Figures and Tables

**Table 1:**
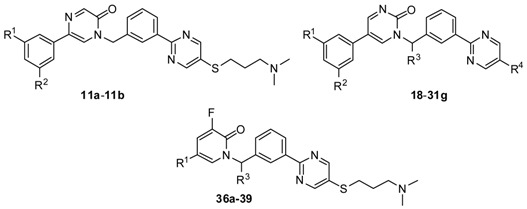
Structures and antiproliferative activity of target compounds.

Comp.	R^1^	R^2^	R^3^	R^4^	IC_50_ of Against 97H cells (µM)
**11a**	CN	H	-	-	9.810 ± 0.790
**11b**	F	F	-	-	9.10 ± 0.670
**18**	CN	H	H	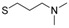	0.443 ± 0.004
**22**	CN	H	H	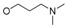	0.559 ± 0.018
**25**	CN	H	H	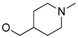	0.361 ± 0.012
**31a**	CN	H	CH_3_	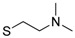	0.186 ± 0.007
**31b**	CN	H	CH_3_	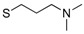	0.149 ± 0.012
**31c**	CN	H	CH_3_	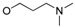	0.216 ± 0.087
**31d**	CN	H	CH_3_	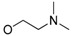	0.196 ± 0.056
**(Rac)-31e**	CN	H	CH_3_	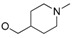	0.026 ± 0.003
**(*R*)-31e**	CN	H	CH_3_	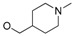	0.018 ± 0.006
**(*S*)-31e**	CN	H	CH_3_	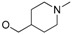	1.834 ± 0.026
**31f**	CN	H	CH_3_	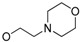	0.248 ± 0.034
**31g**	F	F	CH_3_	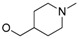	0.332 ± 0.044
**36a**	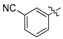	-	H	-	0.149 ± 0.035
**36b**	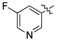	-	H	-	3.807 ± 0.045
**36c**	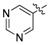	-	H	-	0.671 ± 0.056
**39**	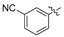	-	CH_3_	-	0.206 ± 0.013
**Tepotinib**	-	-	-	-	0.013 ± 0.008

**Table 2:** The inhibitory activity of compound **31e** against cancer cells (97H, Pc9, Hela, SJSA1), human normal cells (LO2 and HLF), and c-Met enzyme.

IC_50_ (μM)						
Comp.	Pc9	Hela	SJSA1	LO2	HLF	C-Met
**31e**	42.9 ± 0.13	>50	>50	>50	>50	0.032 ± 0.06
**Tepotinib**	8.105 ± 0.012	11.22 ± 0.32	12.17 ± 0.35	10.33 ± 0.46	9.44 ± 0.15	0.004 ± 0.001

## Supplementary Materials

The spectra of ^1^H-NMR and ^13^C-NMR for the target compounds are available online.
