# Supplementary material for: Synthesis, Evaluation, and Mechanism Study of New Tepotinib Derivatives as Antiproliferative Agents
Source: Molecules. 2019 Mar 25;24(6):1173. doi: 10.3390/molecules24061173 (PMC6470795; doi:10.3390/molecules24061173)
Supplement: Supplementary file 1 [file molecules-24-01173-s001.pdf]

Synthesis, evaluation, and mechanism study of new Tepotinib derivatives as antiproliferative agents

Niuniu Zhang, Baijiao An, Yan Zhou, Xingshu Li* and Ming Yan*

Institute of Drug Synthesis and Pharmaceutical Process, School of Pharmaceutical Sciences, Sun Yat-sen University, Guangzhou, China.
Email: yanming@mail.sysu.edu.cn

Supporting Information

1. **Synthesis and Chemical Information Data** 2-9
2. **Biological assay result** 10
3. **Copies of NMR and HPLC spectra** 11-30

Synthesis of compound 2a, 2b

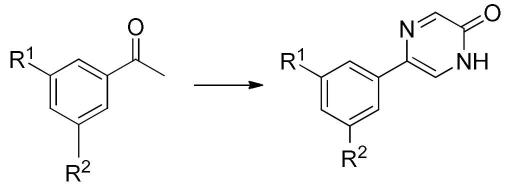

1a: R^1 = CN; R^2 = H
1b: R^1 = R^2 = F

2a: R^1 = CN; R^2 = H
2b: R^1 = R^2 = F

To a stirred mixture of SeO_2 (20 mmol, 2.2 g) in dioxane (20 mL) was added water (2 mL) and heated to reflux until SeO_2 was dissolved. Compound **1a** (10 mmol, 1.45 g) was added and the mixture was stirred at reflux overnight. After completion, the mixture was filtered through a pad of celite. The filtrate was concentrated in vacuo. Compound 3-(2-oxoacetyl)benzonitrile was obtained and used directly in next step without further purification. To a suspension of α-aminoacetamide hydrochloride (1.5 g, 14 mmol) in MeOH (12.5 mL)–water (3.1 mL) was added 12.5 M aqueous NaOH (1.65 mL, 20 mmol) solution at -30 °C, and then a solution of NaOH (543 mg, 14 mmol) in MeOH (6.1 mL) was added to the mixture. A solution of 3-(2-oxoacetyl)benzonitrile in MeOH (11.1 mL) was added to the mixture at -20°C, and then the resulting suspension was stirred for 2h at same temperature, and the stirring continued for 1 h at room temperature. After cooling with ice water, the mixture was acidified with AcOH. Collection of the resulting precipitates by filtration gave **2a** (52%) as a red solid: **^{1}H NMR** (500 MHz, DMSO-d_6) δ 12.65 (bs, 1H), 8.29 (s, 1H), 8.25 – 8.16 (m, 2H), 8.12 (s, 1H), 7.75 (m, 1H), 7.62 (m, 1H).

The compound **2b** was synthesized via a similar procedure of **2a**. **^{1}H NMR** (500 MHz, DMSO-d_6) δ 8.25 (m, 1H), 8.08 (s, 2H), 7.62 – 7.53 (m, 2H), 7.12 (m, 1H).

Synthesis of compound 2-(3-(chloromethyl)phenyl)-5-fluoropyrimidine (6)

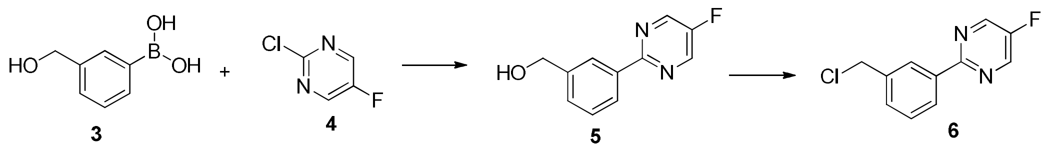

Under nitrogen, a solution of sodium carbonate (4.24g, 40 mmol) in water (20 mL) was added to a solution of 2-chloro-5-fluoropyrimidine **4** (2.47 mL, 20 mmol) in toluene (60 mL), and the mixture was heated to 80 °C. Bis(triphenylphosphine)palladium(II) chloride (701.9 mg, 1 mmol) was added, and a solution of 3-(hydroxymethyl)phenylboronic acid (3.34 g, 22 mmol) in ethanol (20 mL) was subsequently added dropwise. The reaction mixture was stirred at 90 °C for 12 hours. The reaction mixture was cooled to room temperature and filtered. The aqueous phase was extracted with AcOEt (100 mL × 3). The combined organic layer was washed with H_2O (30 mL) and brine (20 mL), and then dried over anhydrous Na_2SO_4, filtered and evaporated in vacuo. The residue was purified by flash

chromatography over silica gel (petroleum/EtOAc = 10:1~3:1) to give (3-(5-fluoropyrimidin-2-yl)phenyl)methanol **5** (68%). **^{1}H NMR** (500 MHz, $CDCl_3$) *δ* 8.61 (s, 2H), 8.32 (s, 1H), 8.25 (m, 1H), 7.50 – 7.40 (m, 2H), 4.74 (s, 2H).

Thionyl chloride (1.06 mL, 15 mmol) was added in portions with stirring to a solution of (3-(5-fluoropyrimidin-2-yl)phenyl)methanol **5** (1.51 g, 3 mmol) in $CHCl_3$ at 0°C. The reaction mixture was stirred at reflux for 3h. The reaction mixture was cooled to room temperature and quenched with saturated aqueous $NaHCO_3$. The aqueous phase was extracted with dichloromethane (80 mL × 2). The combined organic layer was washed with H_2O (80 mL) and brine (60 mL), and then dried over anhydrous Na_2SO_4, filtered and evaporated in vacuo. The residue was purified by flash chromatography over silica gel (petroleum/EtOAc = 20:1~5:1) to give 2-(3-(chloromethyl)phenyl)-5-fluoropyrimidine **6** (599.4 mg, 2.7 mmol, 90%).**^{1}H NMR** (500 MHz, $CDCl_3$) *δ* 8.67 (s, 2H), 8.43 (s, 1H), 8.35 (m, 1H), 7.55 – 7.45 (m, 2H), 4.68 (s, 2H).

Synthesis of compound 7a, 7b

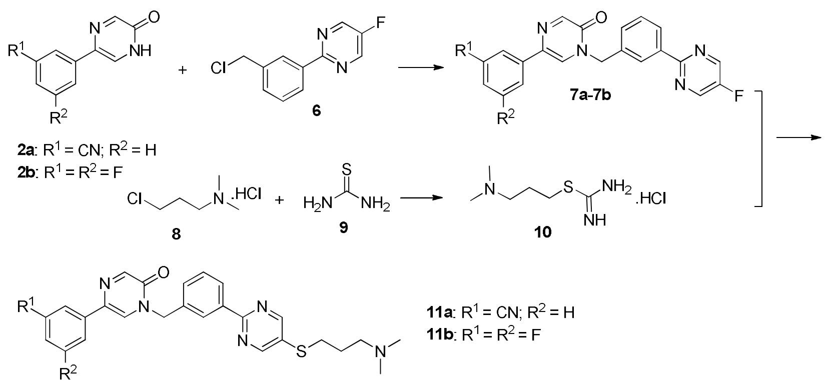

To a suspension of NaH (60% in mineral oil, 44 mg, 1.1 mmol) in DMF (2 mL) at 0°C was added a mixture of 3-(6-oxo-1,6-dihydro-pyridazin-3-yl)-benzonitrile **2a** (197 mg, 1 mmol) in DMF (2 mL) and THF (2 mL). After stirred at 0°C for 30 min, a solution of 2-(3-(chloromethyl)phenyl)-5-fluoropyrimidine **6** (222 mg, 1 mmol) in THF (2 mL) was added. The resulting mixture was stirred at room temperature for 12 h, and cooled with ice bath. The mixture was quenched with 10% aqueous NH_4Cl, and extracted with EtOAc. The combined organic layer was washed with brine, dried Na_2SO_4, filtered and concentrated. The residue was purified by flash chromatography (silica gel, eluted with (petroleum/EtOAc = 10:1~2:1)) to afford the title compound **7a** (153.2 mg, 40%) as green solid. **^{1}H NMR** (400 MHz, $CDCl_3$) *δ* 8.65 (s, 2H), 8.43 (s, 1H), 8.41 – 8.34 (m, 1H), 8.31 (s, 1H), 8.02 (s, 1H), 7.94 (m, 1H), 7.73 (s, 1H), 7.57 (m, 1H), 7.54 – 7.44 (m, 3H), 5.27 (s, 2H). The compound **7b** was synthesized via a similar procedure of **7a**. **^{1}H NMR** (400 MHz, $CDCl_3$) *δ* 8.66 (s, 2H), 8.40 (m, 2H), 8.30 (s, 1H), 7.59 – 7.43 (m, 3H), 7.22 (m, 2H), 6.74 (m, 1H), 5.25 (s, 2H).

Synthesis of compound 17

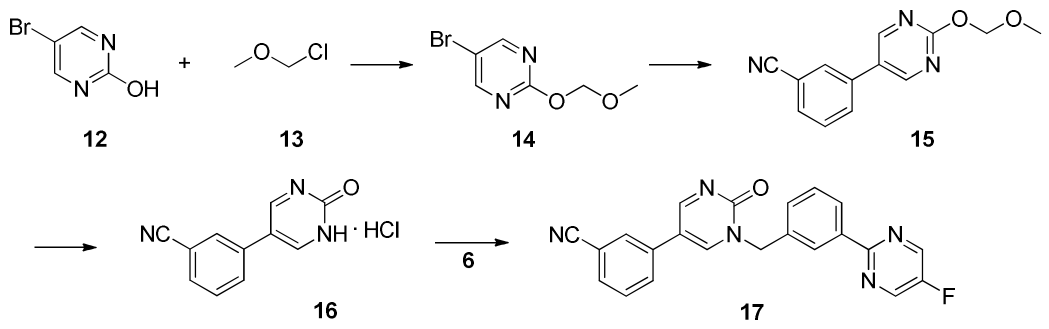

5-bromopyrimidin-2-ol **12** (50 mmol, 8.695 g) was dissolved in 80 mL of dry dichloromethane. Chloromethyl methyl ether **13** (75 mmol, 1.5 eq., 5.7 mL) and *N*, *N*-diisopropylethylamine (110 mmol, 2.2 eq., 19.2 mL) were added at 0°C. Then, the mixture was stirred at room temperature for 6h. The reaction was quenched with an aqueous saturated solution of ammonium chloride (20 mL). The aqueous layer was extracted three times with dichloromethane (3x 100 mL), and the combined organic layers were washed with brine (100 mL), dried over magnesium sulfate and the solvent was removed under reduced pressure. The crude product was purified by column chromatography on a silica gel (petroleum/EtOAc = 1:1~1:2) to give 1-bromo-4-(methoxymethoxy)benzene **14** (5.1 g, 47.4%) as a white solid. **^{1}H NMR** (400 MHz, $CDCl_3$) δ 8.58 (d, *J* = 3.1 Hz, 1H), 7.89 (d, *J* = 3.2 Hz, 1H), 5.25 (s, 2H), 3.46 (s, 3H).

The compound **15** was synthesized via a similar procedure of compound **5**. Compound **15**: Brown solid, yield 65.4%. **^{1}H NMR** (400 MHz, DMSO-d_6) δ 9.10 (d, *J* = 3.3 Hz, 1H), 8.72 (d, *J* = 3.4 Hz, 1H), 8.19 (s, 1H), 8.02 (d, *J* = 8.0 Hz, 1H), 7.81 (m, 1H), 7.66 (m, 1H), 5.27 (s, 2H), 3.37 (s, 3H).

To a solution of the **15** (747 mg, 3.1 mmol) in MeOH (15 mL) at room temperature was added 10% aq. HCl (3.1 mmol). The reaction mixture was heated to reflux for 12h. The reaction mixture was allowed to cool to ambient temperature. The reaction mixture was filtered and the residue was washed by methyl. The white solid compound **16** was obtained without further purified. **^{1}H NMR** (400 MHz, DMSO-d_6) δ 8.62 (s, 1H), 8.04 (s, 1H), 7.90 (d, *J* = 7.9 Hz, 1H), 7.68 (d, *J* = 7.6 Hz, 1H), 7.58 (t, *J* = 7.8 Hz, 1H).

2-(3-(chloromethyl)phenyl)-5-fluoropyrimidine **6** (116.55 mg, 0.525mmol, 1.05eq.) and potassium carbonate (172.5 mg, 1.25 mmol, 2.5eq.) were added to a suspension of **16** (116.75 mg, 0.5 mmol) in dry DMF (10 mL), and the mixture was stirred at 80 °C for 12 hours. The reaction mixture was allowed to cool to room temperature. The aqueous phase was extracted with dichloromethane (20 mL× 3). The combined organic layer was washed with H_2O (10 mL) and brine (20 mL), and then dried over anhydrous Na_2SO_4, filtered and evaporated in vacuo. The residue was purified by flash chromatography over silica gel (DCM/MeOH = 100:1~40:1) to give 3-(1-(3-(5-fluoropyrimidin-2-yl)benzyl)-2-oxo-1,2-dihydropyrimidin-5-yl)benzonitrile **17** white solid (150 mg, 79%) as a white solid. **^{1}H NMR** (500 MHz, $CDCl_3$) δ 8.86 (s, 1H), 8.66 (s, 2H), 8.42 (m, 2H), 7.90 (s, 1H), 7.63 (m, 3H), 7.59 – 7.48 (m, 3H), 5.30 (s, 2H).

Synthesis of compound 21a-21b, 24

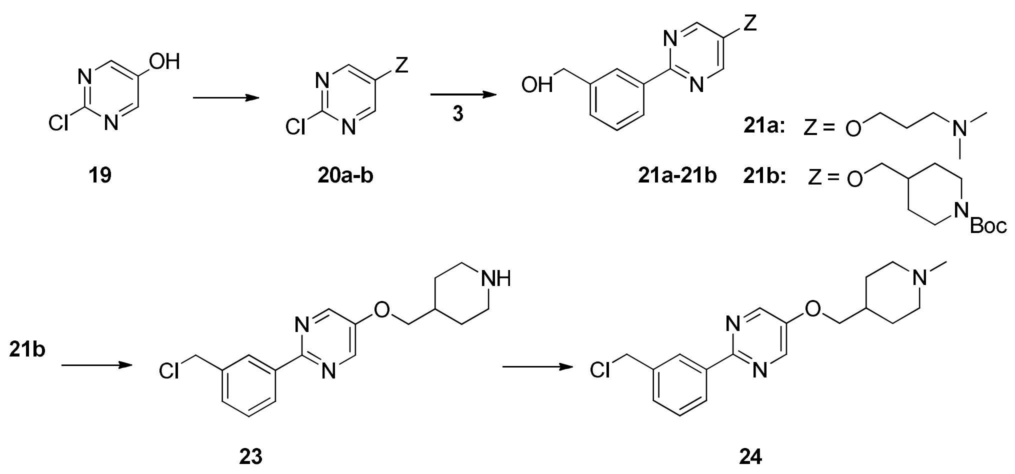

Compound 3-(dimethylamino)propan-1-ol (825.28 mg, 8 mmol) and triphenylphosphine (2.517 g, 9.6 mmol) were added successively to a suspension of **19** (1.044 g, 8 mmol) in THF (15 mL) under nitrogen. A solution of DIAD (1.67 g, 9.6 mmol) in THF (2 mL) was then slowly added dropwise with ice cooling. The resultant solution was stirred at room temperature for 12 hours. The aqueous phase was extracted with dichloromethane (40 mL× 3). The combined organic layer was washed with H_2O (40 mL) and brine (20 mL), and then dried over anhydrous Na_2SO_4, filtered and evaporated in vacuo. The residue was purified by flash chromatography over silica gel (DCM/MeOH = 40:1~10:1) to give **20a.** ^{1}**H NMR** (400 MHz, $CDCl_3$) δ 8.27 (s, 2H), 4.10 (t, *J* = 6.4 Hz, 2H), 2.41 (t, *J* = 6.8 Hz, 1H), 2.22 (s, 6H), 1.95 (m, 2H). The compound **20b** was synthesized via a similar procedure of compound **20a**. ^{1}**H NMR** (500 MHz, $CDCl_3$) δ 8.28 (s, 2H), 4.18 (d, *J* = 13.1 Hz, 2H), 3.90 (d, *J* = 6.0 Hz, 2H), 2.75 (t, *J* = 12.9 Hz, 2H), 2.00 (m, 1H), 1.81 (d, *J* = 13.0 Hz, 2H), 1.47 (s, 9H), 1.29 (m, 2H).

The compound **21a-21b** was synthesized via a similar procedure of compound **5**. **21a**: ^{1}**H NMR** (400 MHz, $CDCl_3$) δ 8.43 (s, 2H), 8.32 (s, 1H), 8.25 (t, *J* = 4.3 Hz, 1H), 7.45 (d, *J* = 4.9 Hz, 1H), 4.76 (s, 2H), 4.12 (t, *J* = 6.3 Hz, 2H), 2.44 (t, *J* = 7.2 Hz, 2H), 2.25 (s, 6H), 2.04 – 1.90 (m, 2H). **21b:** ^{1}**H NMR** (500 MHz, $CDCl_3$) δ 8.47 (s, 2H), 8.39 (s, 1H), 8.36 – 8.28 (m, 1H), 7.48 (m, 2H), 4.68 (s, 2H), 4.20 (bs, 1H), 3.96 (d, *J* = 6.3 Hz, 2H), 2.77 (m, 2H), 2.04 (m, 1H), 1.85 (d, *J* = 12.8 Hz, 2H), 1.46 (s, 9H), 1.41 – 1.31 (m, 2H).

Thionyl chloride (0.6 mL, 7 mmol) was added in portions with stirring to a solution of **21b** (1.37 g, 3.44 mmol) in $CHCl_3$ at 0°C. The reaction mixture was stirred at reflux for 3h. The reaction mixture was cooled to room temperature and quenched with saturated aqueous $NaHCO_3$. The aqueous phase was extracted with dichloromethane (80 mL × 2). The combined organic layer was washed with H_2O (80 mL) and brine (60 mL), and then dried over anhydrous Na_2SO_4, filtered and evaporated in vacuo. The residue was purified by flash chromatography over silica gel (DCM/MeOH = 40:1~10:1) to give 2-(3-(chloromethyl)phenyl)-5-(piperidin-4-ylmethoxy)pyrimidine **23** (874 mg, 2.9 mmol, 85%). ^{1}**H NMR** (500 MHz, DMSO-d_6) δ 8.67 (m, 2H), 8.39 (s, 1H), 8.29 – 8.23 (m, 1H), 7.53 (s, 2H), 4.88 (s, 2H), 4.10 (m, 2H), 3.26 (d, *J* = 11.8 Hz, 1H), 2.86 (m, 2H), 2.17 – 2.07 (m, 1H), 1.95 – 1.87 (m, 2H), 1.58 – 1.46 (m, 2H).

The 2-(3-(chloromethyl)phenyl)-5-(piperidin-4-ylmethoxy)pyrimidine **23** (1 equiv) and was dissolved in methanol (1% acetic acid), formaldehyde (5 equiv, 35% in H_2O) and $NaBH_4$ (2.5 equiv) were added, and the mixture was stirred for 1 h at room temperature. The solvent was evaporated, the residue suspended in satd. Na_2CO_3 solution and extracted three times with CH_2Cl_2. The combined organic layers were dried over Na_2SO_4 and evaporated. The residue was purified by flash chromatography over

silica gel (DCM/MeOH = 40:1~10:1) to give compound **24**. **^{1}H NMR** (500 MHz, $CDCl_3$) δ 8.43 (s, 2H), 8.39 (s, 1H), 8.33 – 8.28 (m, 1H), 7.46 (m, 2H), 4.67 (s, 2H), 3.94 (d, J = 5.8 Hz, 2H), 2.90 (t, J = 13.4 Hz, 2H), 2.30 (s, 3H), 1.97 (t, J = 11.6 Hz, 2H), 1.85 (m, 3H), 1.47 (m, 2H).

Synthesis of compound 30a

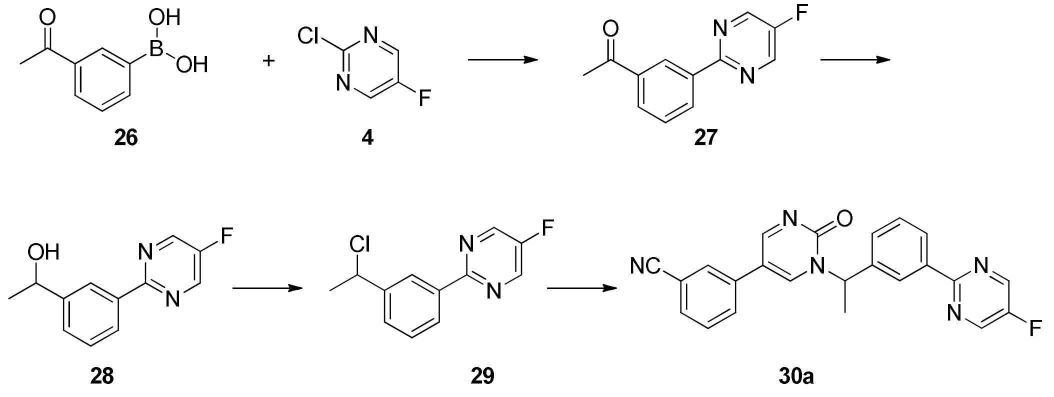

The compound **27** was synthesized via a similar procedure of compound **5.** **^{1}H NMR** (400 MHz, $CDCl_3$) δ 8.97 (s, 1H), 8.67 (s, 2H), 8.58 (d, J = 7.8 Hz, 1H), 8.12 – 8.03 (m, 1H), 7.58 (t, J = 7.8 Hz, 1H), 2.69 (s, 3H).

To a stirred solution of compound **27** (1.3 g, 6.01 mmol) in methanol (20 mL) was added sodium borohydride (272.8 mg, 7.2 mmol) in portions at 0°C. The resulting mixture was warmed to room temperature and stirred for 1 h. Excessive sodium borohydride was then quenched with water (10 mL). The solution was extracted with ethyl acetate (50 mL × 2). The combined organic layer was washed with water (20 mL × 2) and brine (10 mL), dried over anhydrous sodium sulfate, filtered and concentrated in vacuo. The residue was purified by flash chromatography over silica gel (petroleum/EtOAc = 10:1~3:1) to give **28** (867 mg, 66.4%) as a white solid. **^{1}H NMR** (400 MHz, $CDCl_3$) δ 8.61 (s, 2H), 8.34 (s, 1H), 8.24 (d, J = 7.5 Hz, 1H), 7.45 (m, 2H), 4.97 (q, J = 6.5 Hz, 1H), 2.45 (d, J = 5.2 Hz, 1H), 1.53 (d, J = 6.5 Hz, 3H).

The compound **29** was synthesized via a similar procedure of compound **6.**

The compound **30a** was synthesized via a similar procedure of compound **17.** Yellow solid. Yield: 74.6%. **^{1}H NMR** (500 MHz, $CDCl_3$) δ 8.79 (m, 1H), 8.65 (s, 2H), 8.46 (m, 1H), 8.40 (m, 1H), 7.99 (s, 1H), 7.73 (d, J = 3.3 Hz, 1H), 7.60 (m, 1H), 7.55 (m, 1H), 7.51 (m, 3H), 6.36 (q, J = 7.0 Hz, 1H), 1.90 (d, J = 7.0 Hz, 3H).

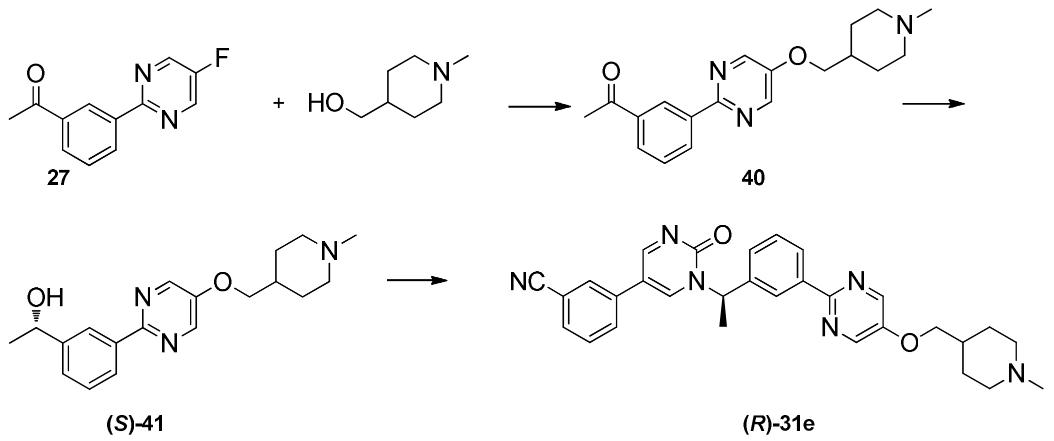

A solution of (1-methylpiperidin-4-yl)methanol (2.3 g, 1.2 eq.) in dry DMF(30 mL) was added to a suspension of NaH (600 mg, 1.5 eq.) in dry DMF (5 mL) at 0°C. After 30 mins, compound **27** (3.2 g, 15mmol, 1 eq.) was added. After the reaction mixture was stirred for 5h, the reaction was quenched with water (20 mL) and the mixture was extracted with dichloromethane (40 mL× 3). The combined organic layer was dried over anhydrous Na_2SO_4 and filtered. After the solvent was removed in vacuo, the crude product was purified by column chromatography (DCM/MeOH = 50:1~10:1) to afford intermediate compound **40** 1-(3-(5-((1-methylpiperidin-4-yl)methoxy)pyrimidin-2-yl)phenyl)ethanone (Yield: 90%) as a yellow solid. **^{1}H NMR** (400 MHz, $CDCl_3$) *δ* 8.94 (t, *J* = 1.6 Hz, 1H), 8.63 – 8.52 (m, 1H), 8.48 (s, 2H), 8.14 – 7.97 (m, 1H), 7.57 (t, *J* = 7.8 Hz, 1H), 3.97 (d, *J* = 5.9 Hz, 2H), 2.94 (m, 2H), 2.71 (s, 3H), 2.32 (s, 3H), 2.09 – 1.95 (m, 2H), 1.86 (m, 3H), 1.65 – 1.42 (m, 2H).

Under nitrogen, a solution of (*S, S*)-CsDPEN (21.3 mg, 0.05 mmol) and dichloro(p-cymene)ruthenium(II) dimer(15.3 mg, 0.005 mmol) in water (2.6 mL) were stirred at 40 °C for 4h, then a solution of CH_3COONa(1.7 g, 25 mmol) in water (10 mL) and 1-(3-(5-((1-methylpiperidin-4-yl)methoxy)pyrimidin-2-yl)phenyl)ethanone(382 mg, 5mmol) in DCM were added to the solution. After the reaction mixture was stirred for 12h, the reaction was quenched with water (40mL) and the mixture was extracted with dichloromethane (40 mL× 3). The combined organic layer was dried over anhydrous Na_2SO_4 and filtered. After the solvent was removed in vacuo, the crude product was purified by column chromatography (DCM/MeOH = 50:1~10:1) to afford intermediate compound **(*S*) -41** (*S*)-1-(3-(5-((1-methylpiperidin-4-yl)methoxy)pyrimidin-2-yl)phenyl)ethanol as a white solid. **^{1}H NMR** (500 MHz, DMSO-*d_6*) *δ* 8.63 (s, 2H), 8.32 (s, 1H), 8.15 (m, 1H), 7.42 (d, *J* = 4.7 Hz, 1H), 5.27 (d, *J* = 3.5 Hz, 1H), 4.91 – 4.73 (m, 1H), 4.05 (d, *J* = 5.8 Hz, 2H), 2.80 (m, 2H), 2.17 (s, 3H), 1.88 (t, *J* = 11.4 Hz, 2H), 1.76 (m, 3H), 1.35 (m, 5H).

The white solid of **(*R*) -41** was synthesized as **(*S*) -41** by (*R, R*)-CsDPEN . **^{1}H NMR** (500 MHz, DMSO-*d_6*) *δ* 8.63 (s, 2H), 8.31 (s, 1H), 8.14 (m, 1H), 7.42 (m, 1H), 5.27 (d, *J* = 3.5 Hz, 1H), 4.87 – 4.70 (m, 1H), 4.04 (d, *J* = 5.8 Hz, 2H), 2.79 (m, 2H), 2.16 (s, 3H), 1.87 (t, *J* = 11.4 Hz, 2H), 1.75 (m, 3H), 1.34 (m, 5H).

The **(*R*)-31e** and **(*S*)-31e** were synthesized as **31e. (*R*)-31e:** Yellow solid. m.p. 97.6-99.4°C. **^{1}H NMR** (500 MHz, DMSO-*d_6*) *δ* 9.03 (d, *J* = 3.2 Hz, 1H), 8.71 (d, *J* = 3.2 Hz, 1H), 8.63 (s, 2H), 8.32 (s, 1H), 8.23 (m, 2H), 8.03 (d, *J* = 8.0 Hz, 1H), 7.81 (d, *J* = 7.7 Hz, 1H), 7.65 (t, *J* = 7.8 Hz, 1H), 7.51 (m, 2H), 6.01 (q, *J* = 7.0 Hz, 1H), 4.03 (d, *J* = 5.8 Hz, 2H), 2.79 (d, *J* = 11.1 Hz, 2H), 2.16 (s, 3H), 1.91 (d, *J* = 7.2 Hz, 3H), 1.86 (d, *J* = 11.3 Hz, 2H), 1.74 (m, 3H), 1.31 (q, 2H). **^{13}C NMR** (101 MHz, $CDCl_3$) *δ* 163.97, 156.82, 155.49, 151.98,

143.94, 142.52, 138.66, 134.92, 131.44, 130.30, 130.17, 129.77, 129.44, 129.34, 128.21, 126.52, 118.26, 116.75, 113.71, 73.39, 56.42, 55.30, 46.35, 35.26, 28.81, 19.21. **HRMS (ESI)** calculated for $C_{30}H_{30}N_6O_2$ $[M+H]^+$: 507.2503, found: 507.2482. Purity: 98.1% (by HPLC).

(*S*)-31e: Yellow solid. m.p. 98.0-99.6°C. **^{1}H NMR** (500 MHz, DMSO-*d*$_6$) δ 9.04 (d, *J* = 3.2 Hz, 1H), 8.72 (d, *J* = 3.2 Hz, 1H), 8.64 (s, 2H), 8.33 (s, 1H), 8.23 (m, 2H), 8.03 (d, *J* = 8.0 Hz, 1H), 7.81 (d, *J* = 7. 7 Hz, 1H), 7.65 (t, *J* = 7.8 Hz, 1H), 7.52 (m, 2H), 6.02 (q, *J* = 7.0 Hz, 1H), 4.04 (d, *J* = 5.8 Hz, 2H), 2.80 (d, *J* = 11.1 Hz, 2H), 2.16 (s, 3H), 1.92 (d, *J* = 7.2 Hz, 3H), 1.87 (d, *J* = 11.3 Hz, 2H), 1.74 (m, 3H), 1.32 (q, 2H). **^{13}C NMR** (101 MHz, $CDCl_3$) δ 163.97, 156.82, 155.49, 151.98, 143.94, 142.52, 138.66, 134.92, 131.44, 130.30, 130.17, 129.77, 129.44, 129.34, 128.21, 126.52, 118.26, 116.75, 113.71, 73.39, 56.42, 55.30, 46.35, 35.26, 28.81, 19.21. **HRMS (ESI)** calculated for $C_{30}H_{30}N_6O_2$ $[M+H]^+$: 507.2503, found: 507.2496. Purity: 96.2% (by HPLC).

Synthesis of compound 35a-c

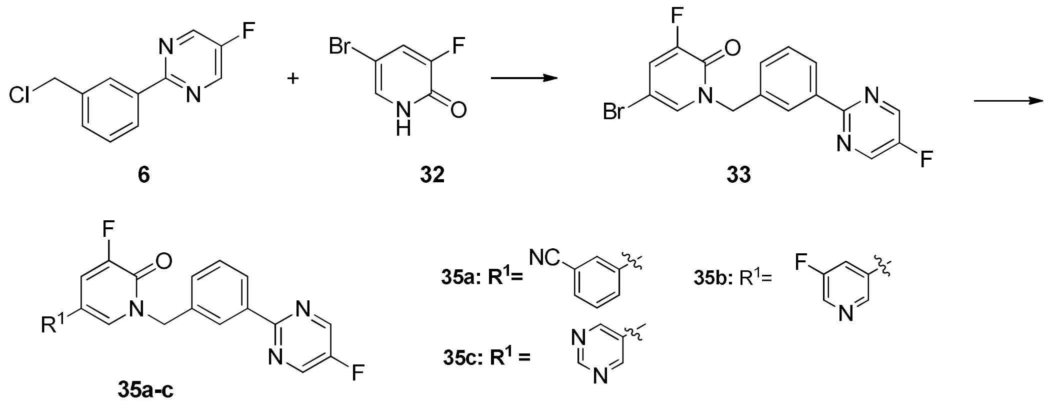

2-(3-(chloromethyl)phenyl)-5-fluoropyrimidine **6** (1.1 g, 5 mmol) and potassium carbonate (1.03 g, 7.5 mmol, 1.5eq.) were added to a suspension of 5-bromo-3-fluoropyridin-2(1H)-one **32** (955 mg, 5 mmol) in dry DMF (20 mL), and the mixture was stirred at 80 °C for 12 hours. The reaction mixture was allowed to cool to room temperature. The aqueous phase was extracted with dichloromethane (50 mL× 3). The combined organic layer was washed with H_2O (30 mL) and brine (20 mL), and then dried over anhydrous Na_2SO_4, filtered and evaporated in vacuo. The residue was purified by flash chromatography over silica gel (petroleum/EtOAc = 10:1~2:1) to give 5-bromo-3-fluoro-1-(3-(5-fluoropyrimidin-2-yl)benzyl)pyridin-2(1H)-one **33** (1.5 g, 79.7%) as a white solid. **^{1}H NMR** (500 MHz, $CDCl_3$) δ 8.66 (s, 2H), 8.46 – 8.28 (m, 2H), 7.54 – 7.39 (m, 2H), 7.29 – 7.27 (m, 1H), 7.18 (m, 1H), 5.24 (s, 2H).

Under nitrogen, a solution of sodium carbonate (414 mg, 3 mmol) in water (1 mL) was added to a solution of 5-bromo-3-fluoro-1-(3-(5-fluoropyrimidin-2-yl)benzyl)pyridin-2(1H)-one **33** (377 mg, 1 mmol) in DME (9 mL), and the mixture was heated to 80 °C. Bis(triphenylphosphine)palladium(II) chloride (57 mg, 0.05 mmol) was added, and a solution of (3-cyanophenyl)boronic acid (220.5 mg, 1.5 mmol, 1.5eq) in DMF (0.5 mL) was subsequently added dropwise. The reaction mixture was stirred at 89 °C for 12 hours. The reaction mixture was cooled to room temperature and filtered. The aqueous phase was extracted with dichloromethane (20 mL× 3). The combined organic layer was washed with H_2O (10 mL) and brine (10 mL), and then dried over anhydrous Na_2SO_4, filtered and evaporated in

vacuo. The residue was purified by flash chromatography over silica gel (petroleum/EtOAc = 5:1~1:1) to give 3-(5-fluoro-1-(3-(5-fluoropyrimidin-2-yl)benzyl)-6-oxo-1,6-dihydropyridin-3-yl)benzonitrile **35a** (340 mg, 85%) as a gray solid. **^{1}H NMR** (500 MHz, $CDCl_3$) δ 8.66 (s, 2H), 8.41 (s, 1H), 8.37 (t, *J* = 4.4 Hz, 1H), 7.62 – 7.52 (m, 4H), 7.50 (m, 2H), 7.41 (s, 1H), 7.36 (m, 1H), 5.36 (s, 2H).

The compound **35b-c** was synthesized via a similar procedure of **35a.35b: ^{1}H NMR** (400 MHz, $CDCl_3$) δ 8.65 (s, 2H), 8.49 – 8.32 (m, 4H), 7.50 (m, 2H), 7.43 (s, 1H), 7.36 (m, 2H), 5.36 (s, 2H).

35c: **^{1}H NMR** (400 MHz, DMSO-d_6) δ 9.13 (s, 1H), 9.10 (s, 2H), 8.98 (s, 2H), 8.53 (s, 1H), 8.40 (s, 1H), 8.27 (d, *J* = 7.7 Hz, 1H), 8.14 – 8.04 (m, 1H), 7.58 (m, 1H), 7.53 (m, 1H), 5.33 (s, 2H).

Synthesis of compound 38

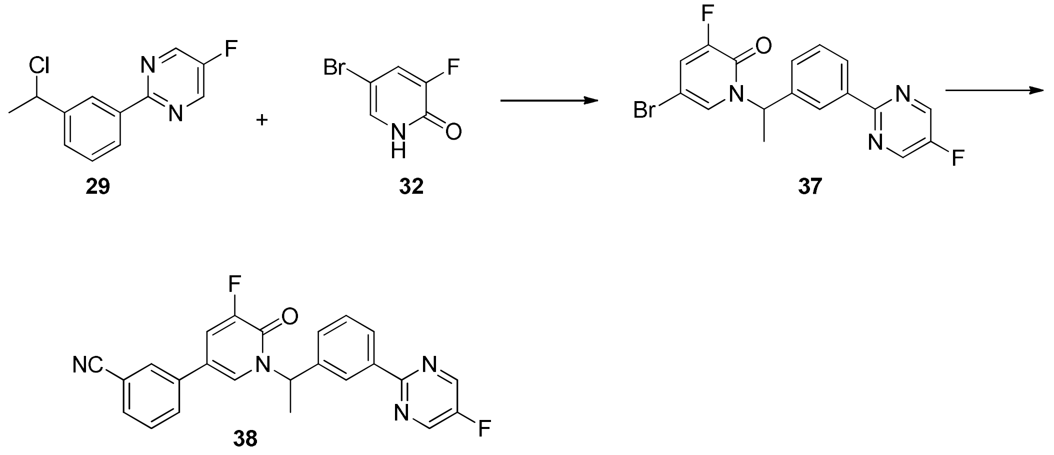

The compound **37** was synthesized via a similar procedure of compound **33.** Yield**:** 32.6%. White solid. **^{1}H NMR** (400 MHz, $CDCl_3$) δ 8.67 (s, 2H), 8.40 (m, 2H), 7.52 (m, 1H), 7.46 – 7.37 (m, 1H), 7.14 (m, 1H), 7.10 – 7.03 (m, 1H), 6.55 – 6.48 (q, 1H), 1.82 (d, *J* = 7.1 Hz, 3H).

The compound **38** was synthesized via a similar procedure of compound **35a. ^{1}H NMR** (400 MHz, $CDCl_3$) δ 8.67 (s, 2H), 8.49 (m, 1H), 8.43 – 8.33 (m, 1H), 7.47 (m, 6H), 7.37 – 7.26 (m, 1H), 7.24 – 7.17 (m, 1H), 6.68 – 6.55 (q, *J* = 7.1 Hz, 1H), 1.89 (d, *J* = 7.1 Hz, 3H).

Selectivity of **31e** toward other cancer cells and human normal cells.

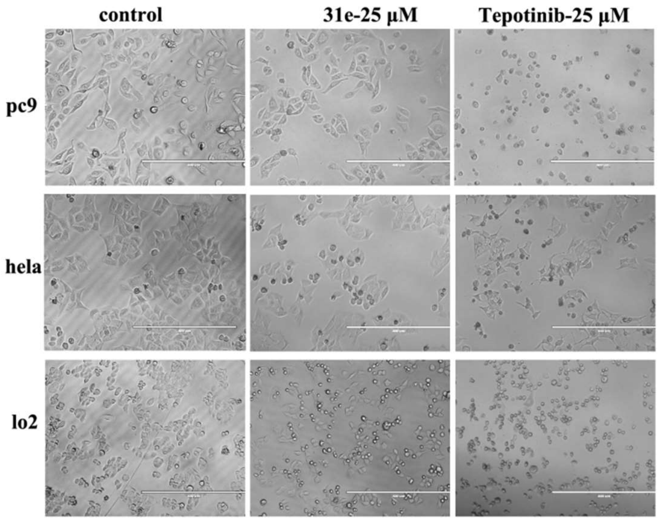

Figure 1. The human cancer cell lines (Pc9, Hela) and human normal cell lines (LO_2) were treated with **31e** or **Tepotinib** at the indicated concentration or DMSO (0.01%) for 48 h, the detection of the fixed cells morphology was performed with microscope. All images were collected with a 10×objective.

11a

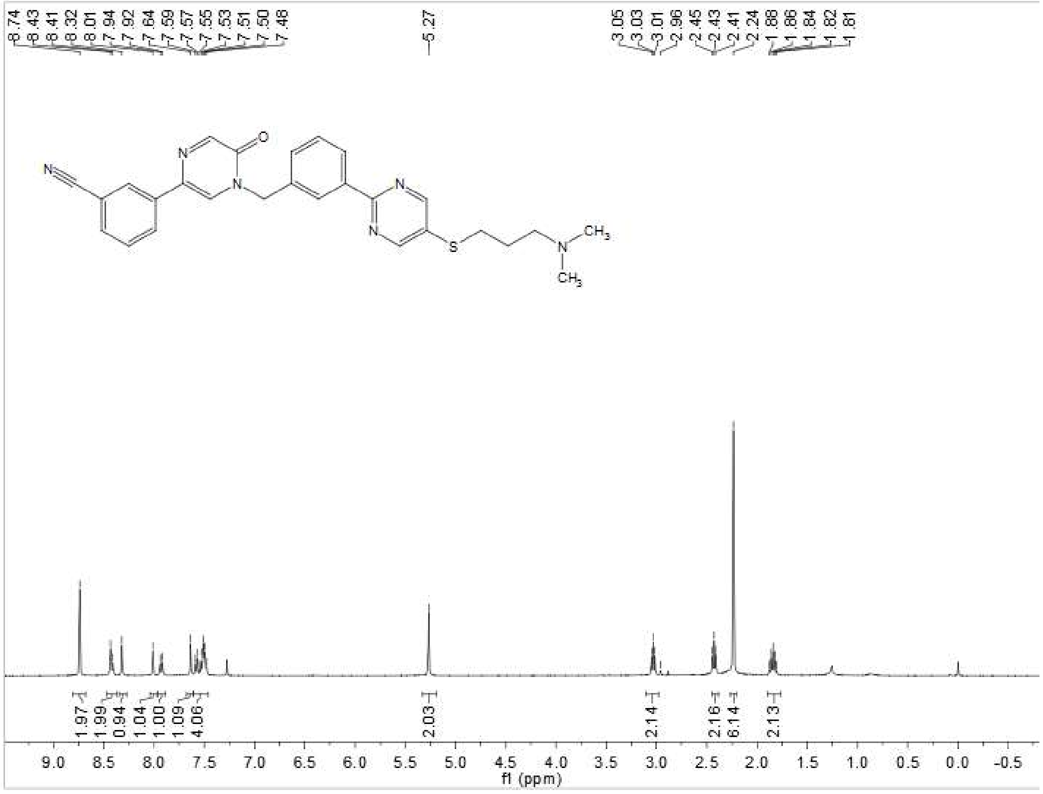

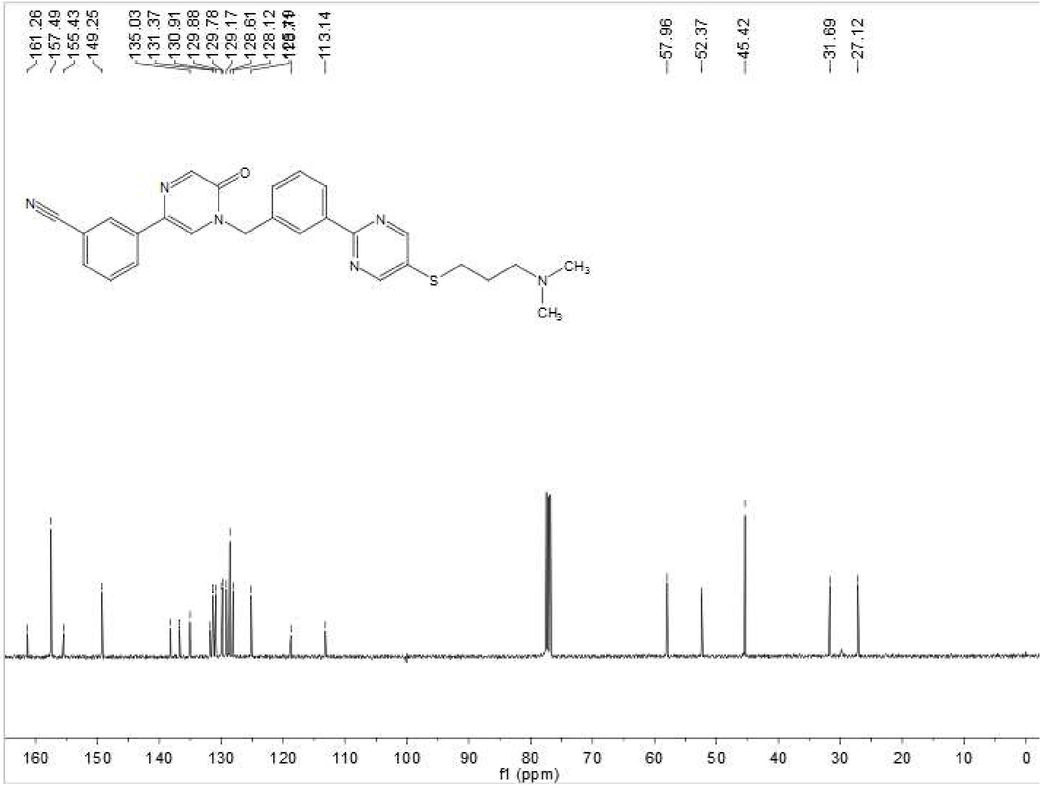

11b

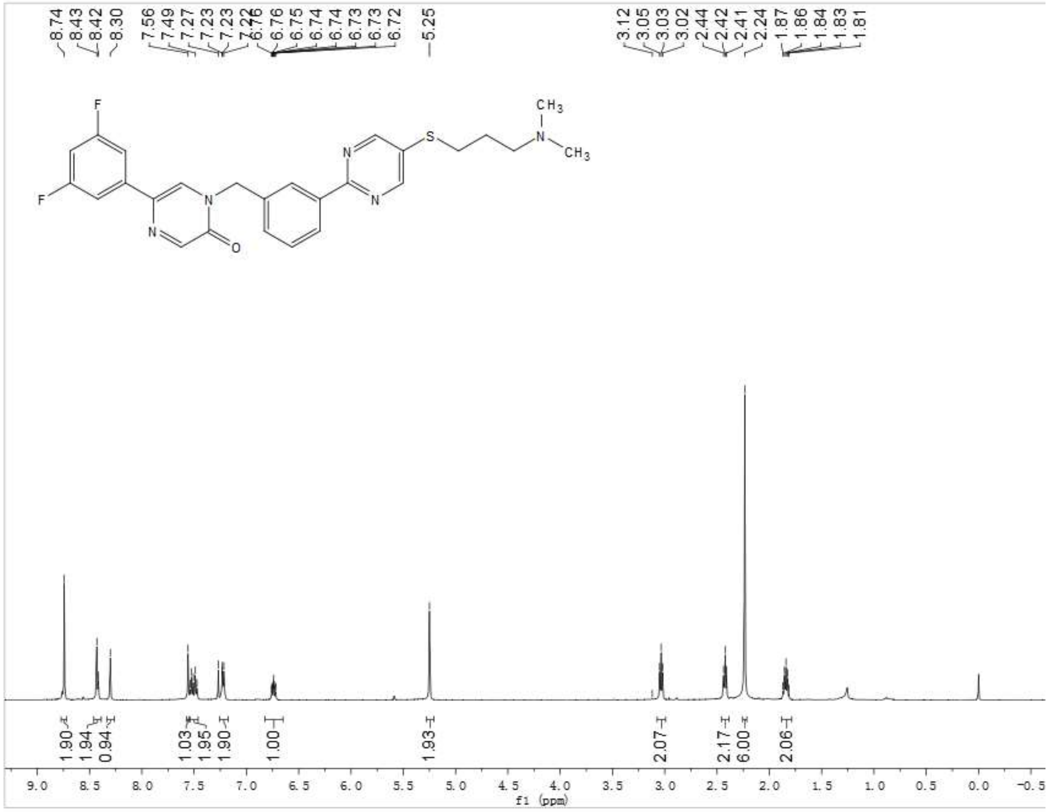

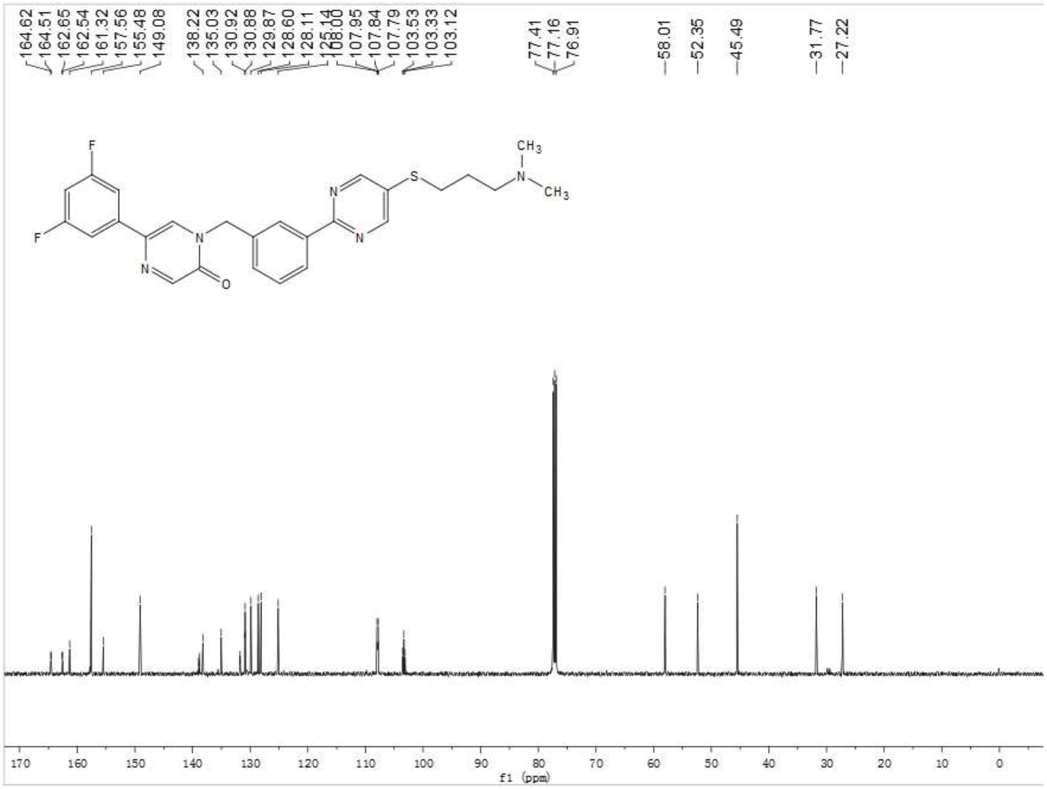

18

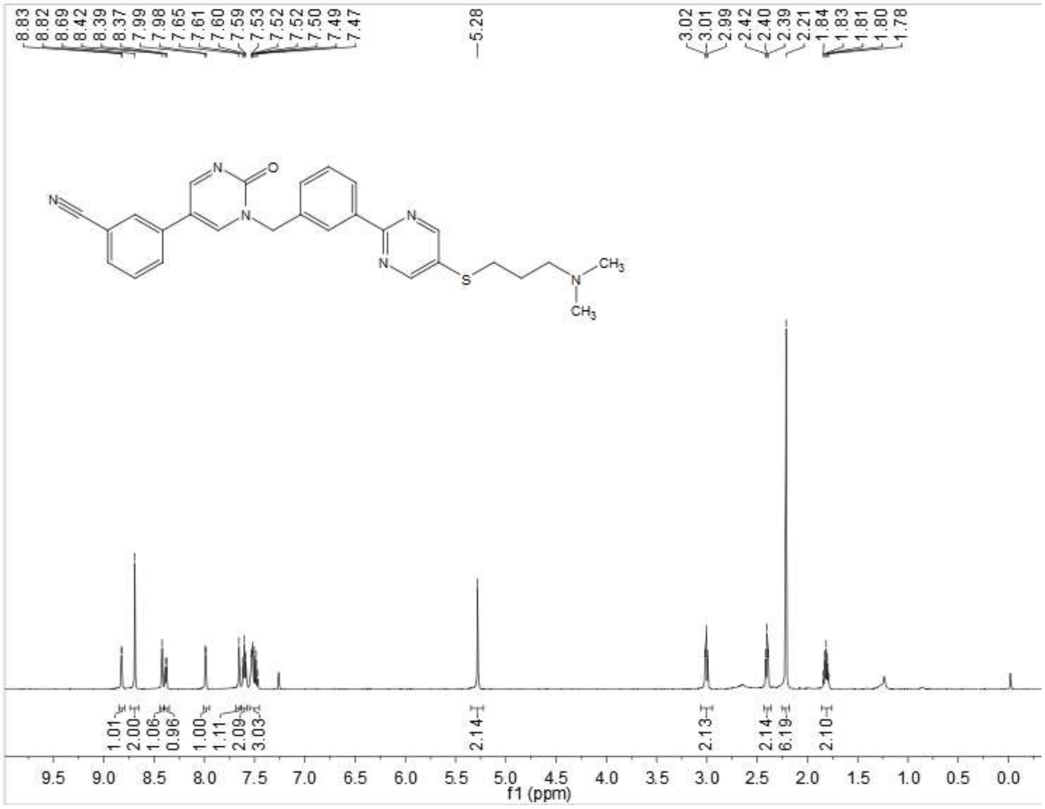

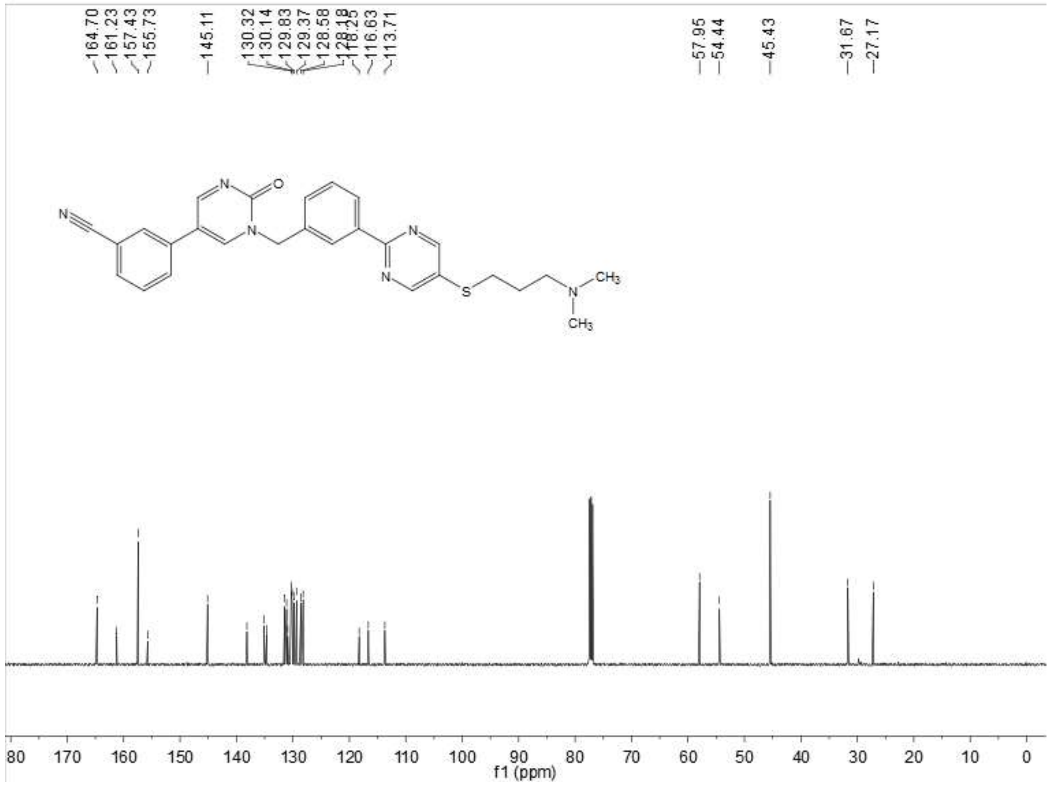

22

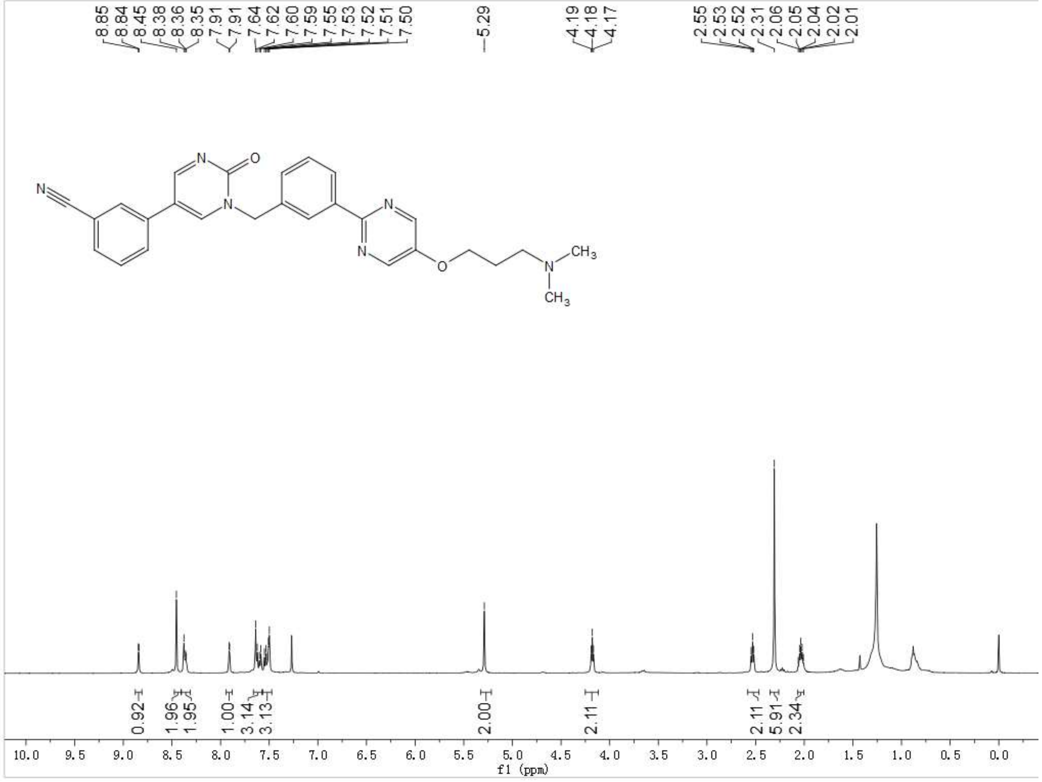

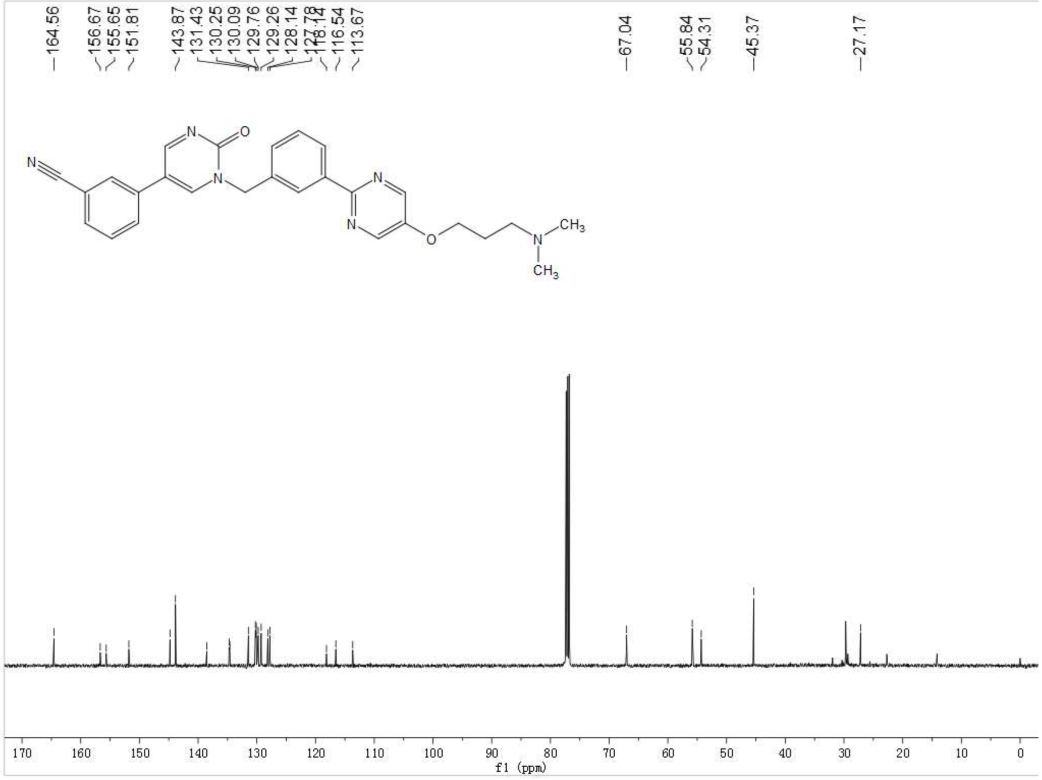

25

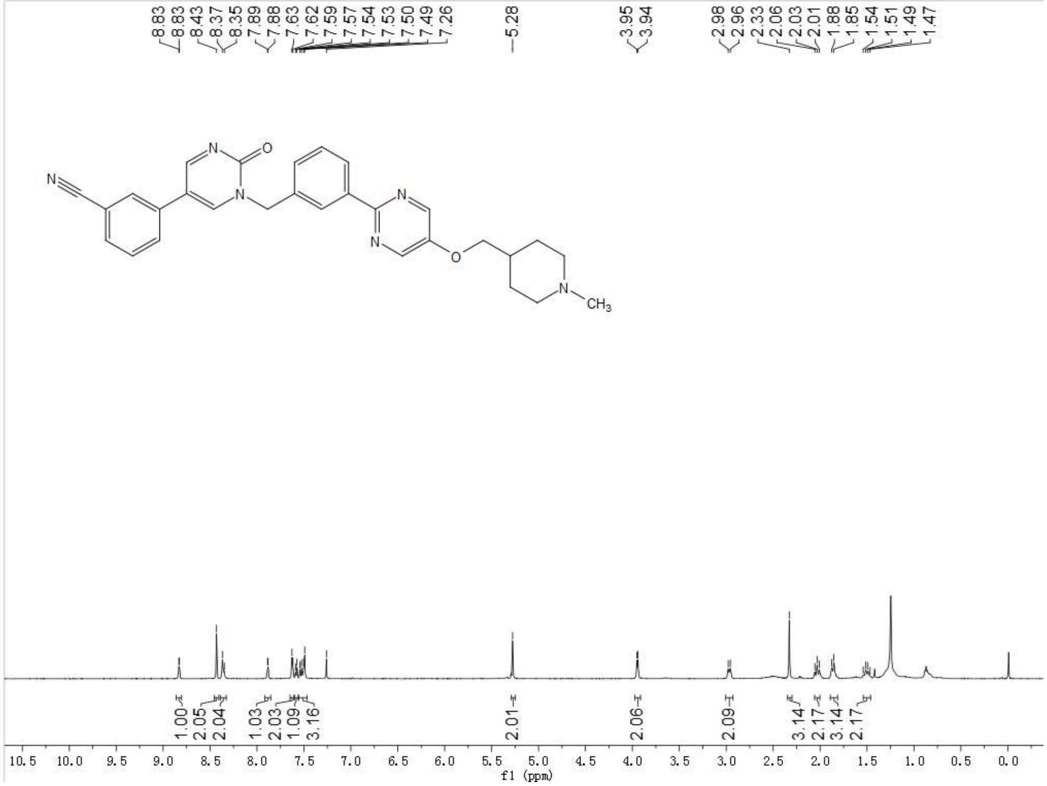
8.83 8.83 8.43 8.37 8.35 7.89 7.88 7.63 7.62 7.59 7.57 7.54 7.53 7.50 7.49 7.26
5.28
3.95 3.94
2.98 2.96 2.33 2.06 2.03 2.01 1.88 1.85 1.54 1.51 1.49 1.47
N
O
N
N
N
N
O
N
CH_3
1.00 2.05 2.04 1.03 2.03 1.09 3.16
2.01
2.06
2.09
3.14 2.17 3.14 2.17
10.5 10.0 9.5 9.0 8.5 8.0 7.5 7.0 6.5 6.0 5.5 5.0 4.5 4.0 3.5 3.0 2.5 2.0 1.5 1.0 0.5 0.0
f1 (ppm)

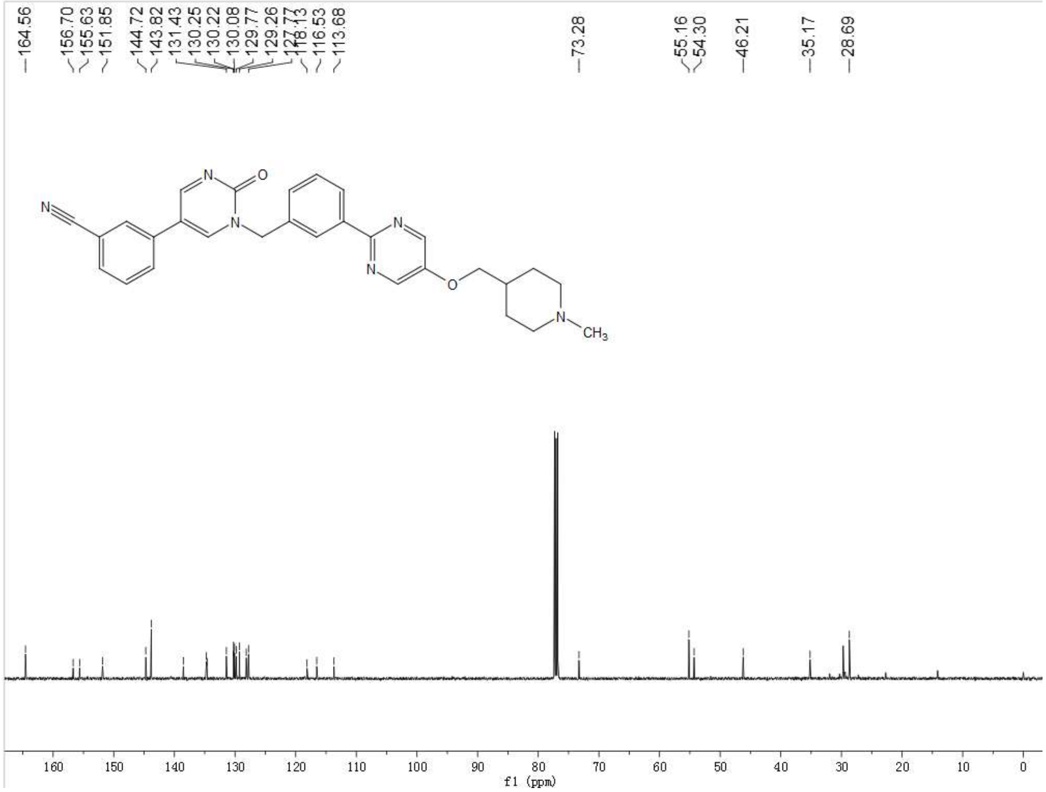
164.56
156.70 155.63 151.85
144.72 143.82 131.43 130.25 130.22 130.08 129.77 129.26 127.77 118.13 116.53 113.68
73.28
55.16 54.30
46.21
35.17
28.69
N
O
N
N
N
N
O
N
CH_3
160 150 140 130 120 110 100 90 80 70 60 50 40 30 20 10 0
f1 (ppm)

31a

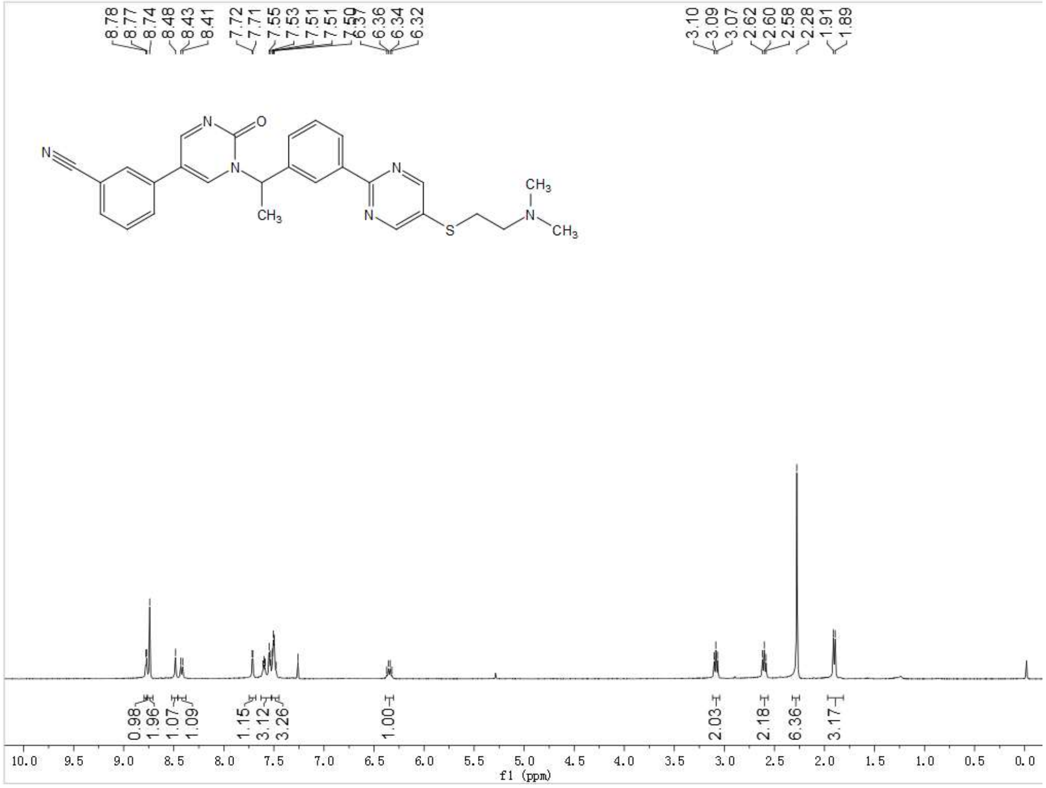

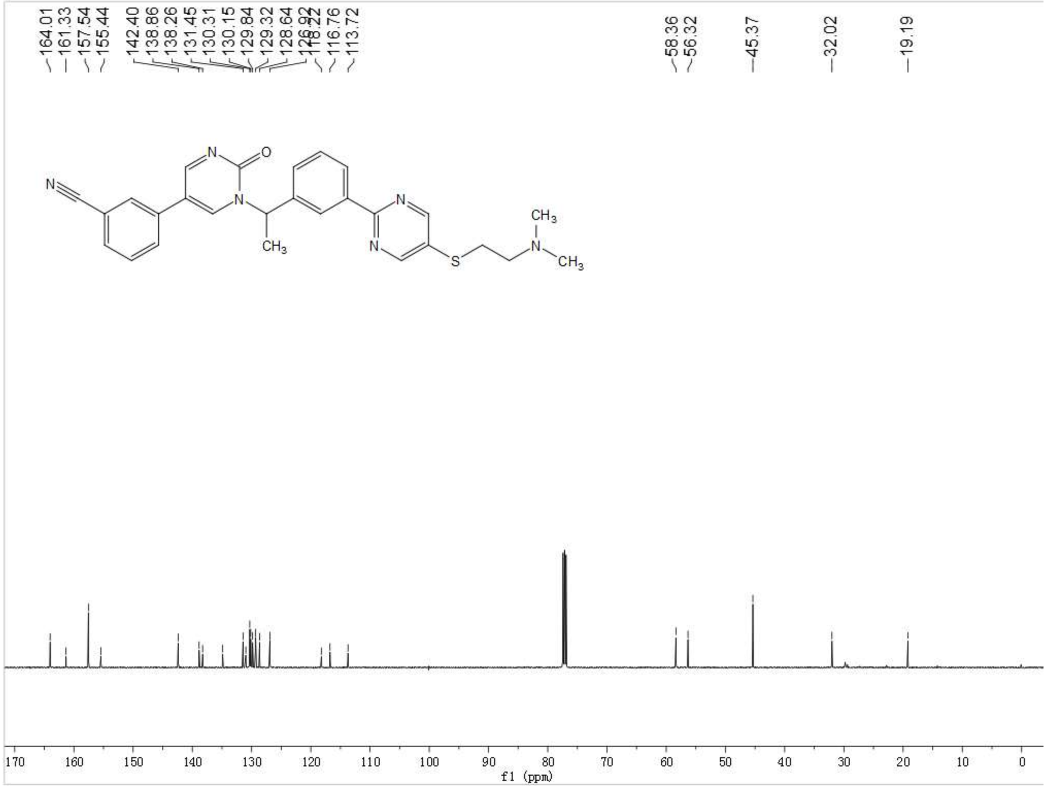

31b

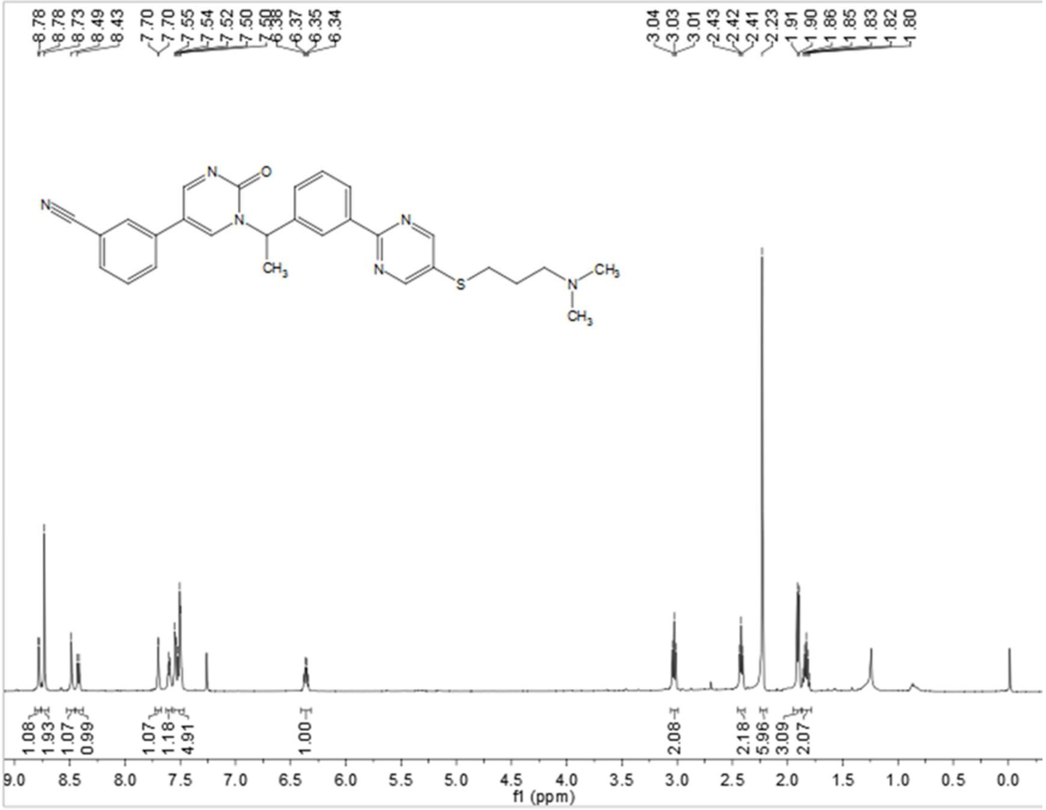

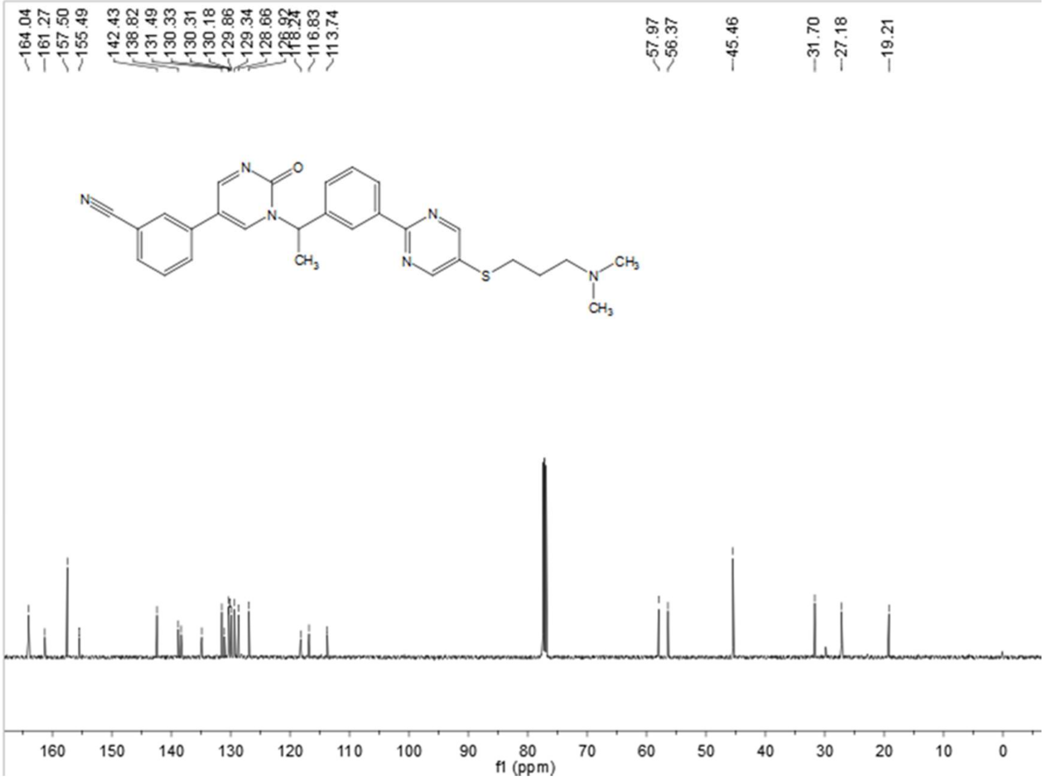

31c

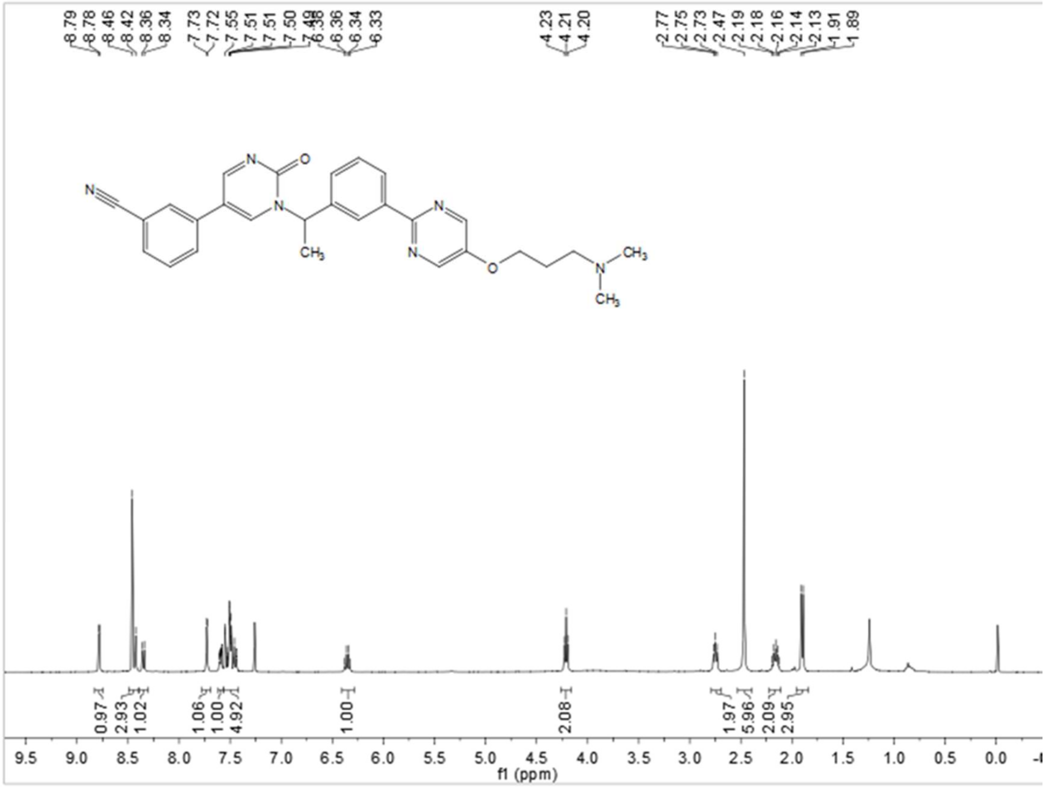
8.79
8.78
8.46
8.42
8.36
8.34
7.73
7.72
7.55
7.51
7.51
7.50
6.36
6.34
6.33
4.23
4.21
4.20
2.77
2.75
2.73
2.47
2.19
2.18
2.16
2.14
2.13
1.91
1.89
N
O
CH3
0.97
2.93
1.02
1.06
1.00
4.92
1.00
2.08
1.97
5.96
2.09
2.95
f1 (ppm)

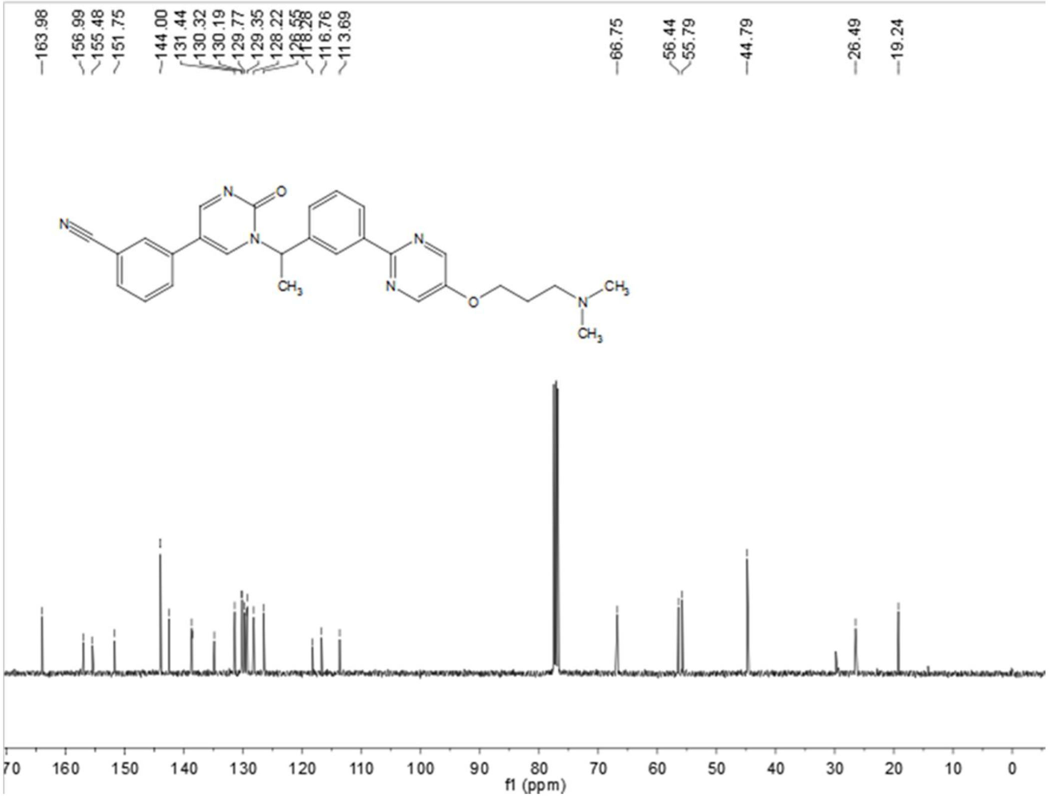
163.98
156.99
155.48
151.75
144.00
131.44
130.32
130.19
129.77
129.35
128.22
116.76
113.69
66.75
56.44
55.79
44.79
26.49
19.24
N
O
CH3
f1 (ppm)

31d

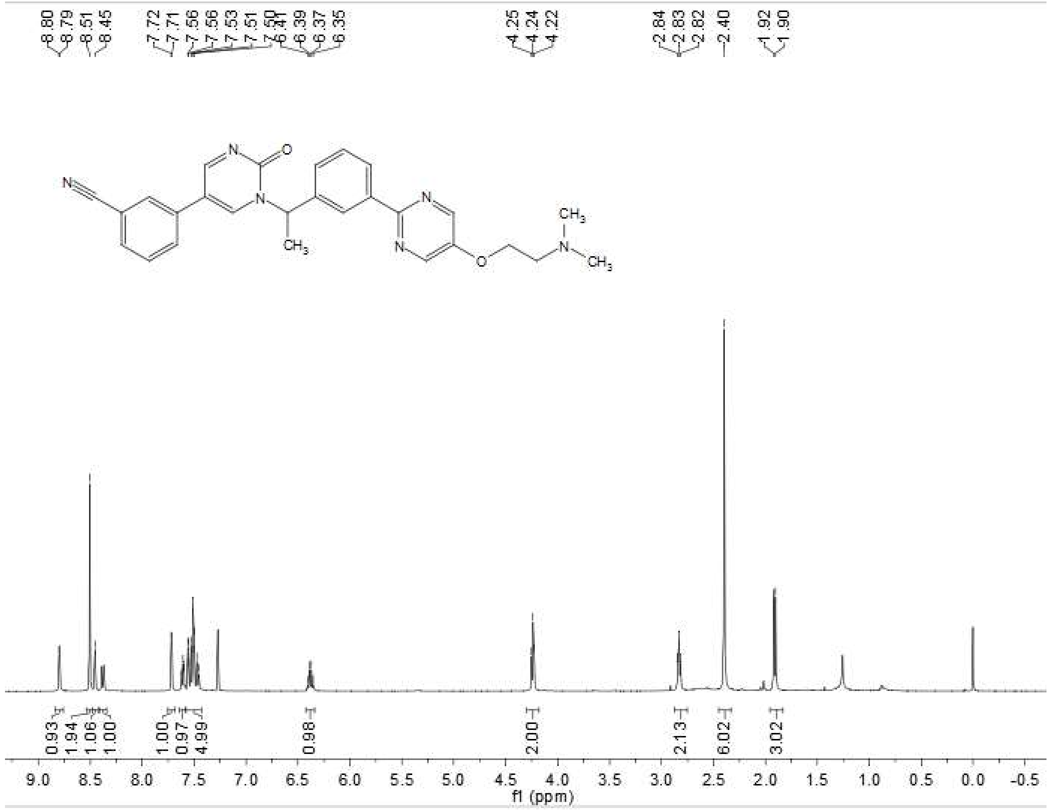
8.80
8.79
8.51
8.45
7.72
7.71
7.56
7.53
7.51
7.50
6.41
6.39
6.37
6.35
4.25
4.24
4.22
2.84
2.83
2.82
2.40
1.92
1.90
N
N
O
N
N
N
O
N
CH3
CH3
CH3
0.93
1.94
1.06
1.00
1.00
0.97
4.99
0.98
2.00
2.13
6.02
3.02
9.0 8.5 8.0 7.5 7.0 6.5 6.0 5.5 5.0 4.5 4.0 3.5 3.0 2.5 2.0 1.5 1.0 0.5 0.0 -0.5
f1 (ppm)

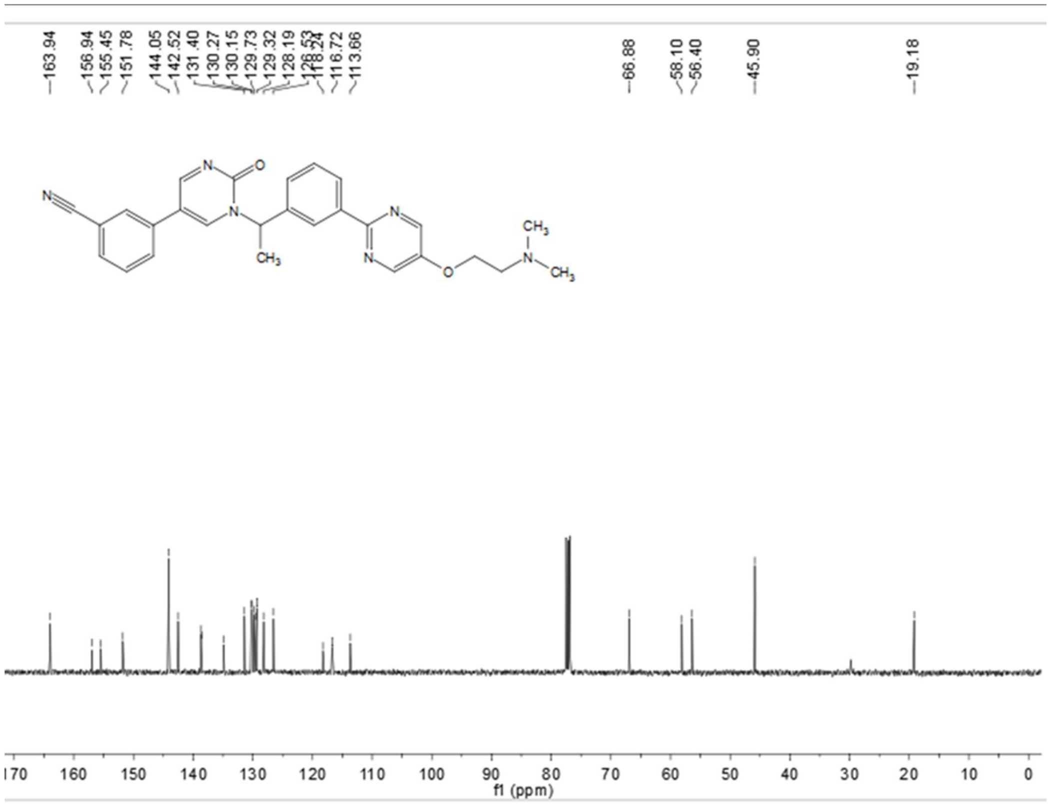
163.94
156.94
155.45
151.78
144.05
142.52
131.40
130.27
130.15
129.73
129.32
128.19
126.53
118.24
116.72
113.66
66.88
58.10
56.40
45.90
19.18
N
N
O
N
N
N
O
N
CH3
CH3
CH3
170 160 150 140 130 120 110 100 90 80 70 60 50 40 30 20 10 0
f1 (ppm)

31e

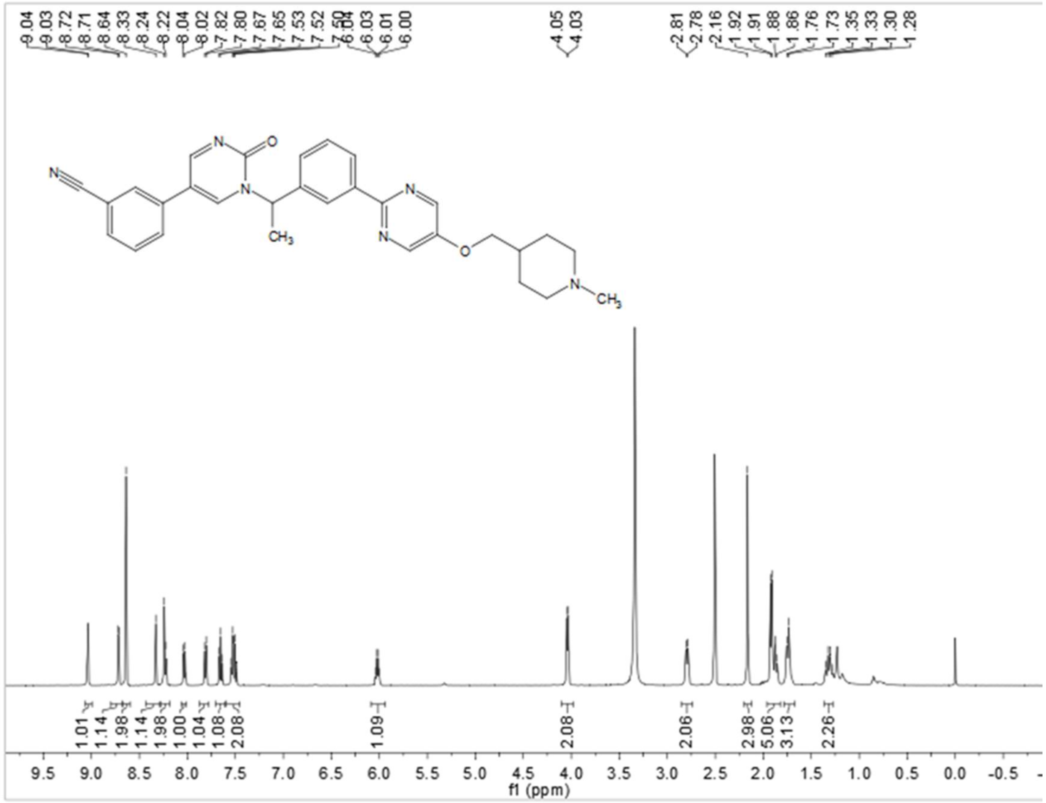

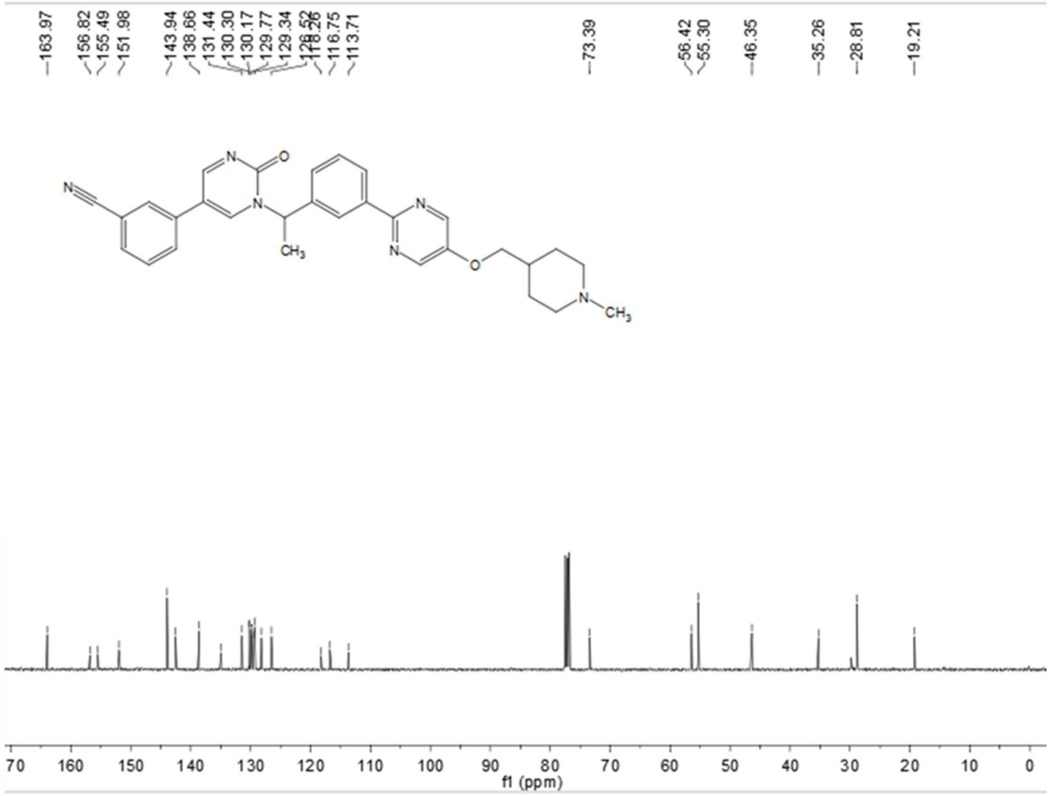

31f

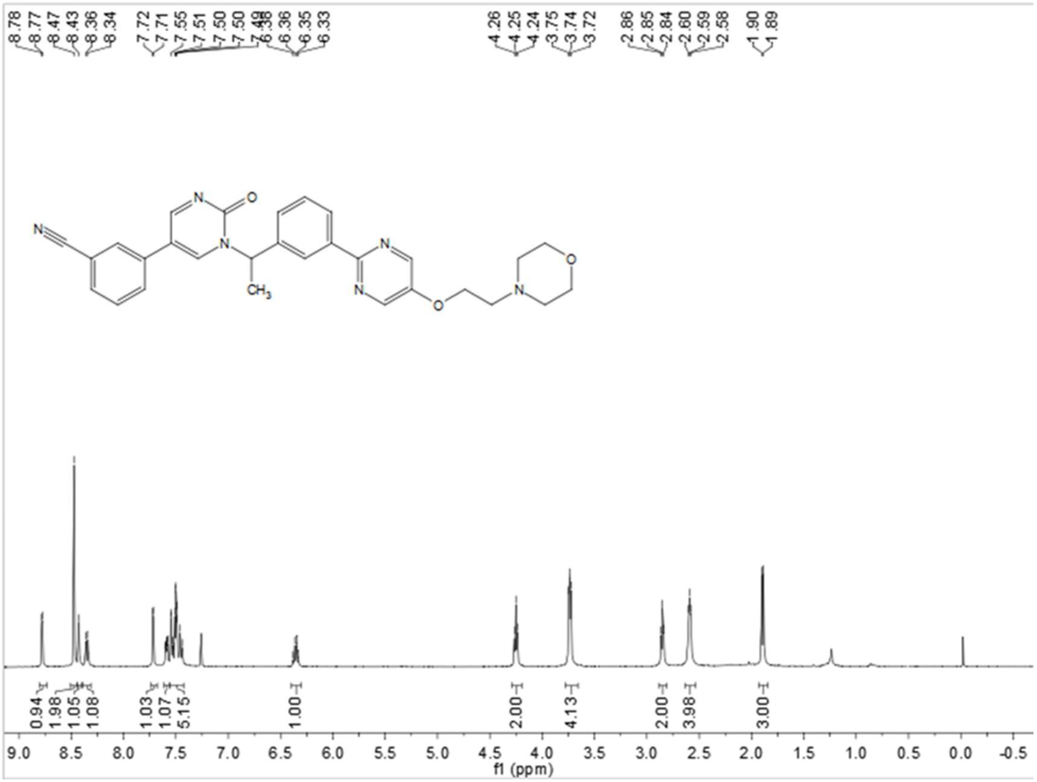
8.78
8.77
8.47
8.43
8.36
8.34
7.72
7.71
7.55
7.51
7.50
7.50
6.36
6.35
6.33
4.26
4.25
4.24
3.75
3.74
3.72
2.86
2.85
2.84
2.60
2.59
2.58
1.90
1.89
N
O
N
N
CH3
N
N
O
N
O
0.94
1.98
1.05
1.08
1.03
1.07
5.15
1.00
2.00
4.13
2.00
3.98
3.00
9.0 8.5 8.0 7.5 7.0 6.5 6.0 5.5 5.0 4.5 4.0 3.5 3.0 2.5 2.0 1.5 1.0 0.5 0.0 -0.5
f1 (ppm)

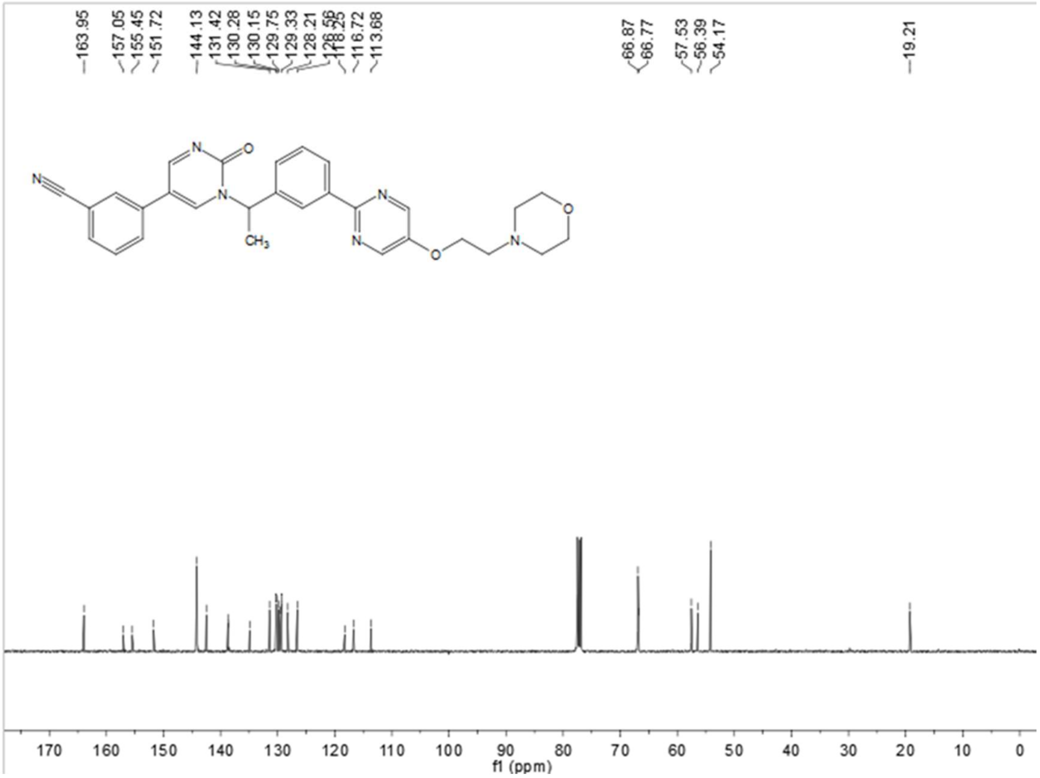
163.95
157.05
155.45
151.72
144.13
131.42
130.28
130.15
129.75
129.33
128.21
116.72
113.68
66.87
66.77
57.53
56.39
54.17
19.21
N
O
N
N
CH3
N
N
O
N
O
170 160 150 140 130 120 110 100 90 80 70 60 50 40 30 20 10 0
f1 (ppm)

31g

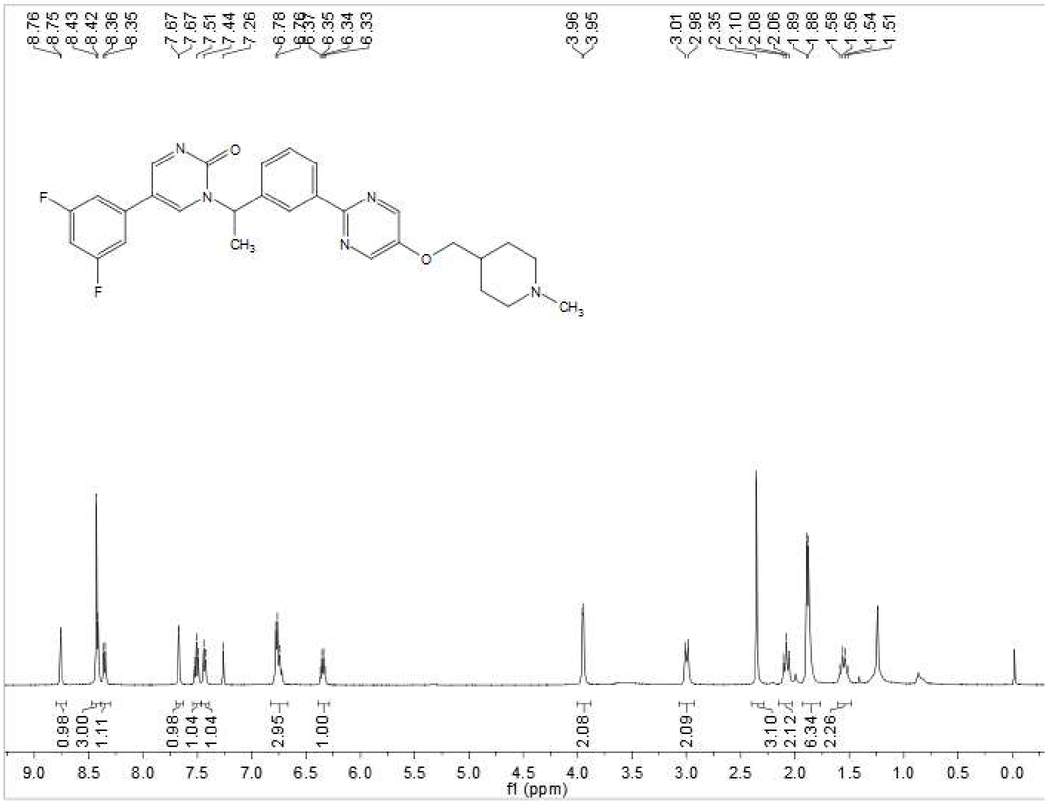

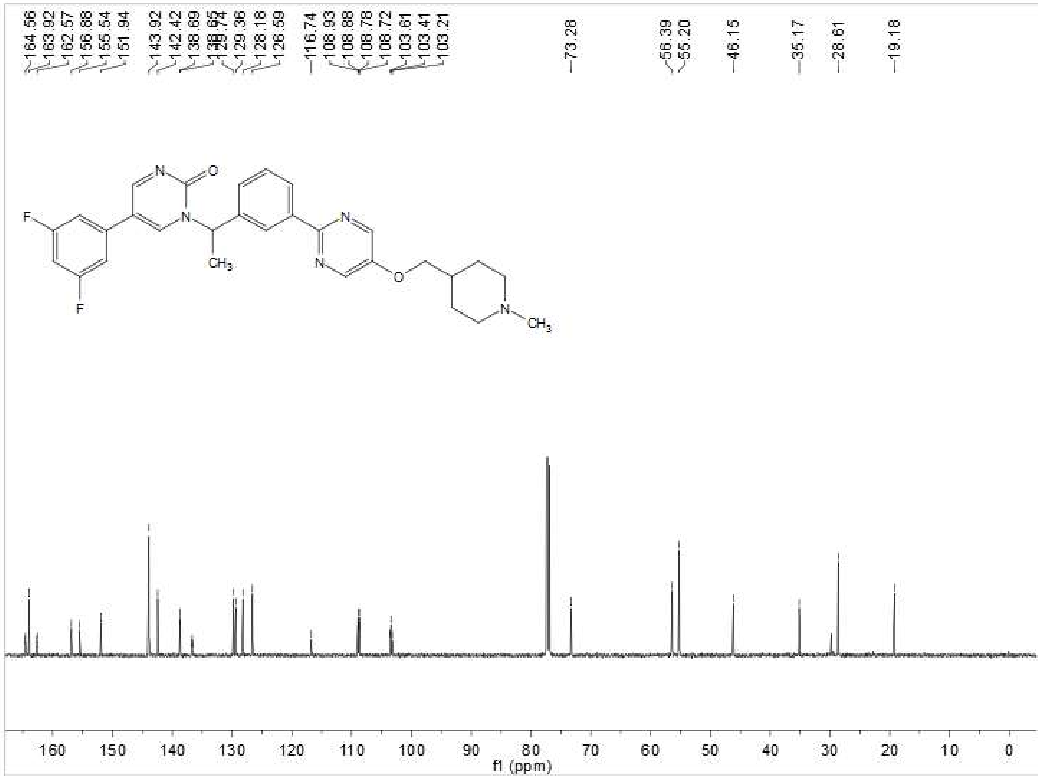

36a

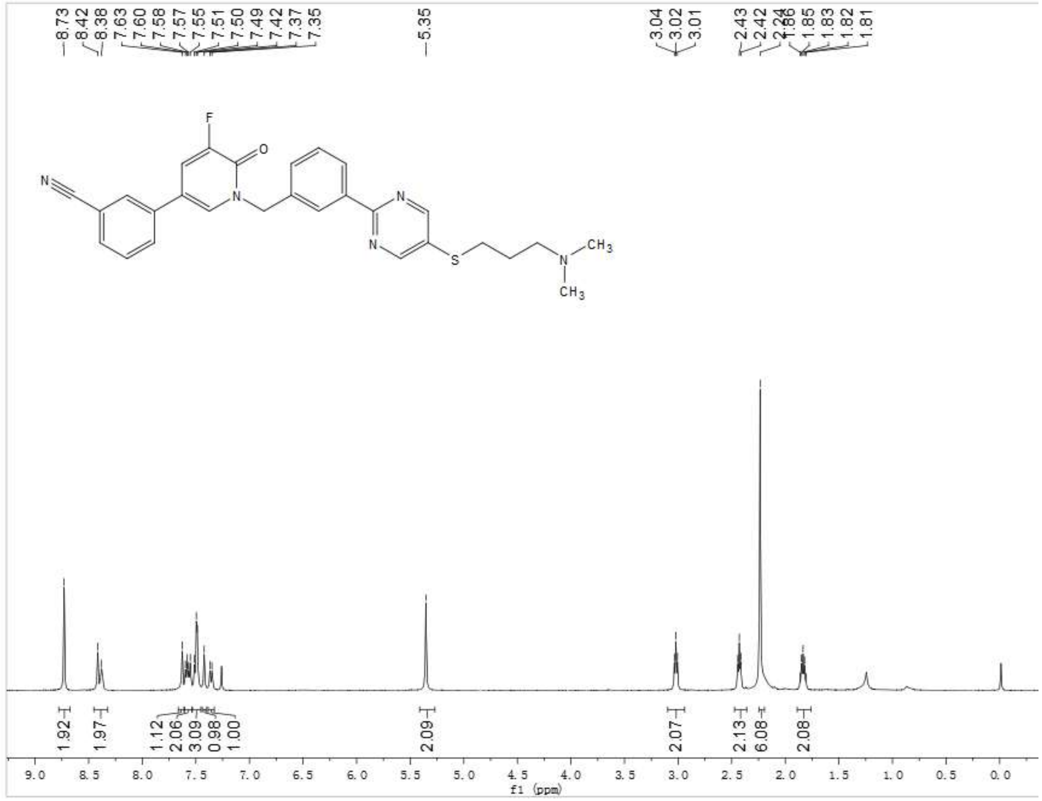

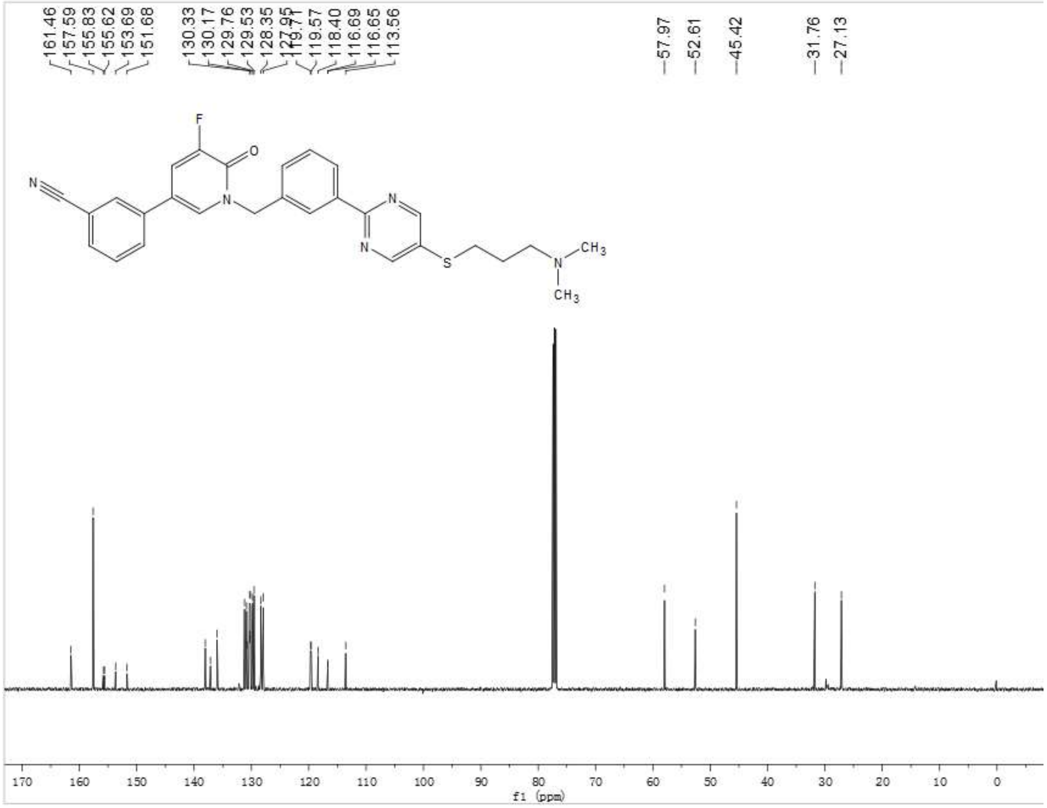

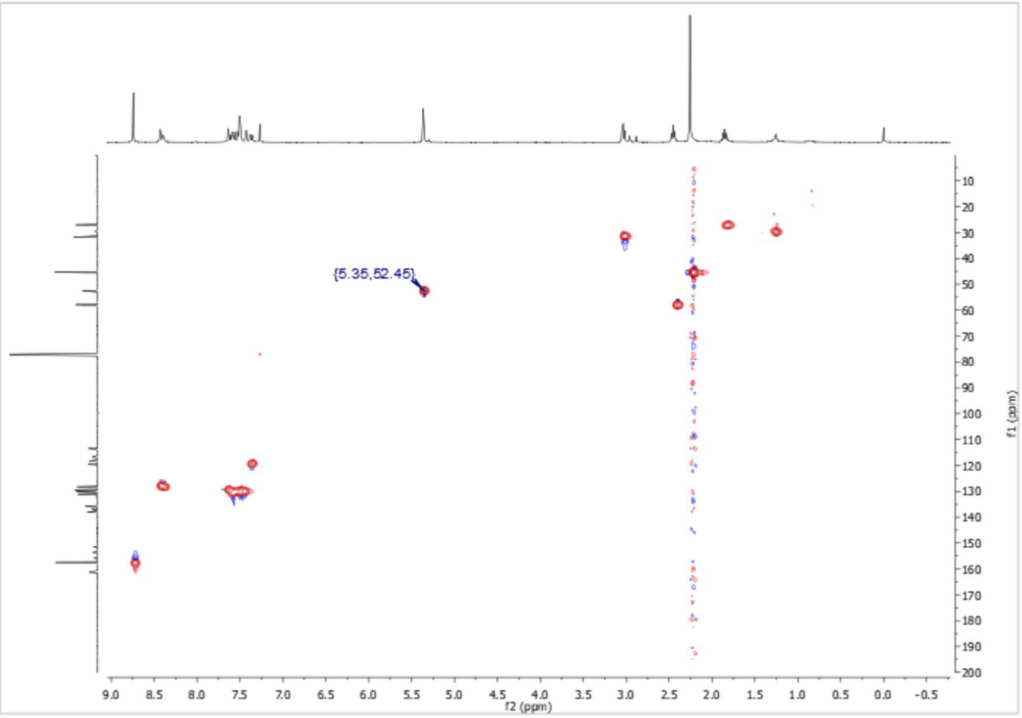
{5.35,52.45}
f2 (ppm)
f1 (ppm)

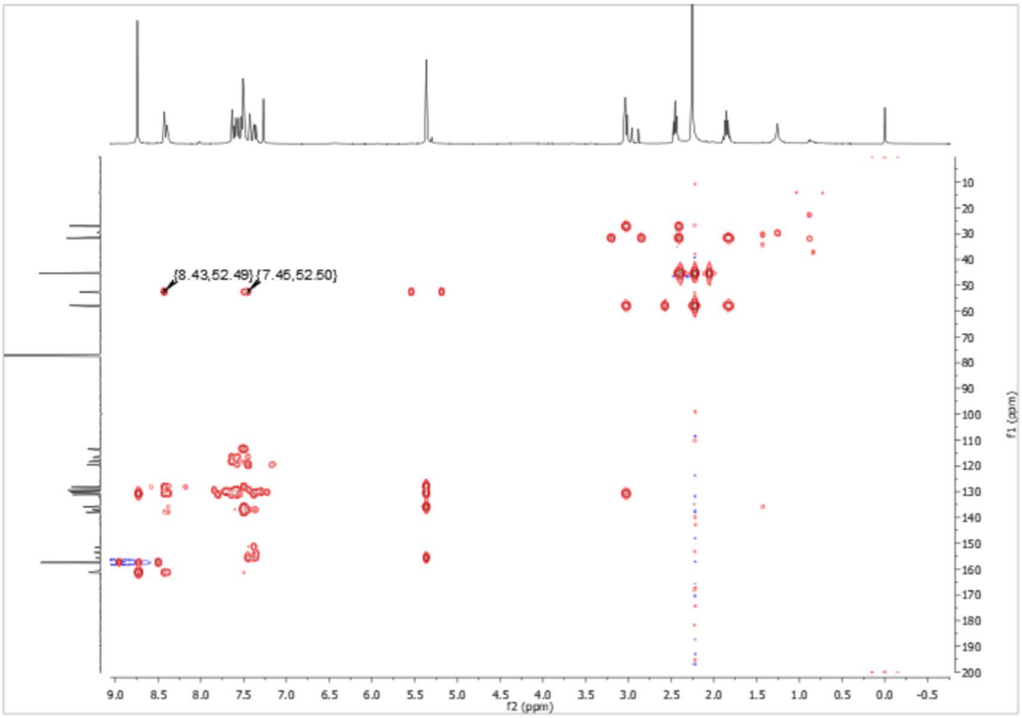
{8.43,52.49}
{7.45,52.50}
f2 (ppm)
f1 (ppm)

36b

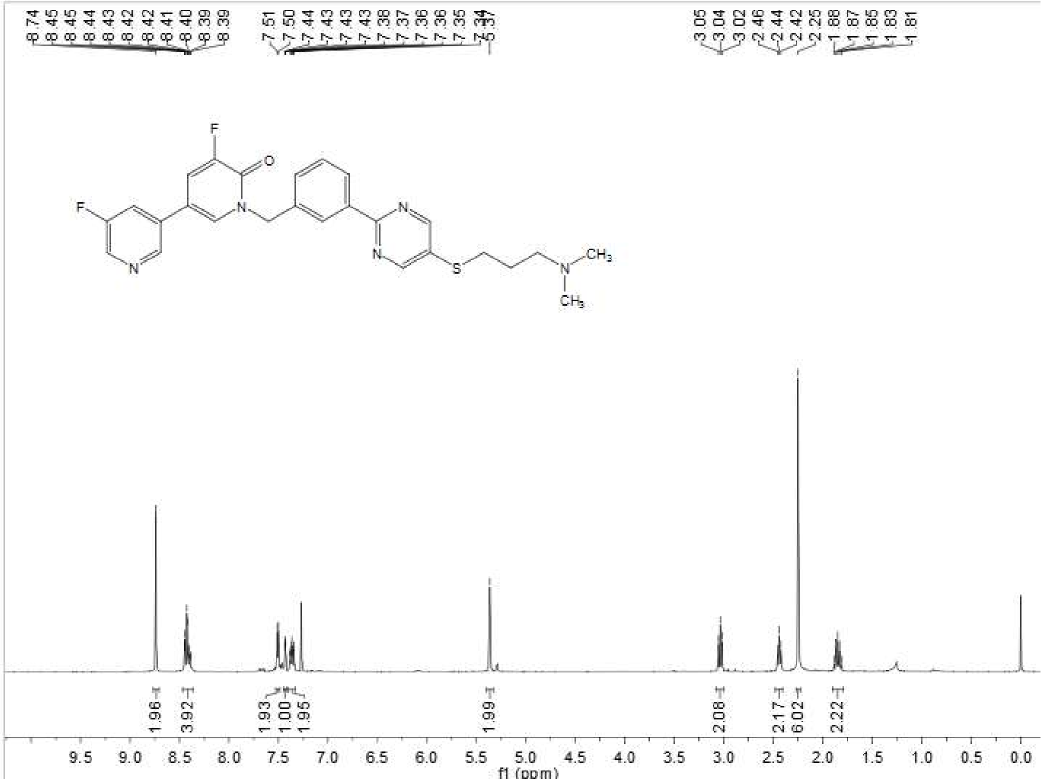

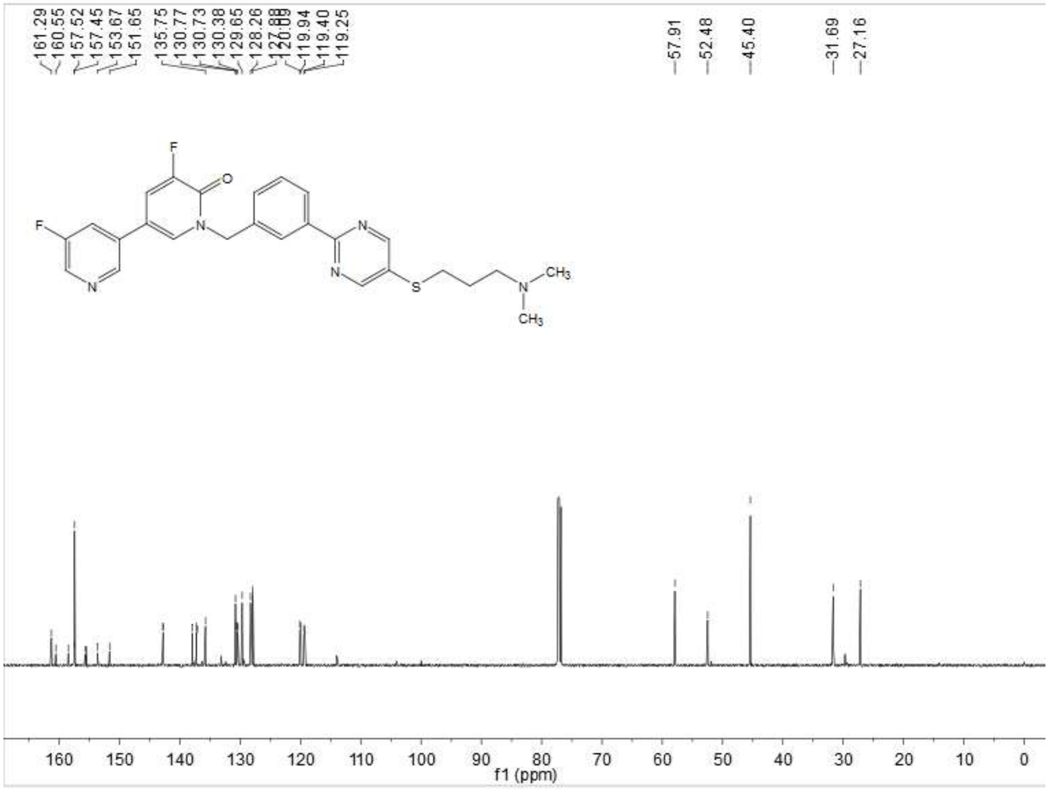

36c

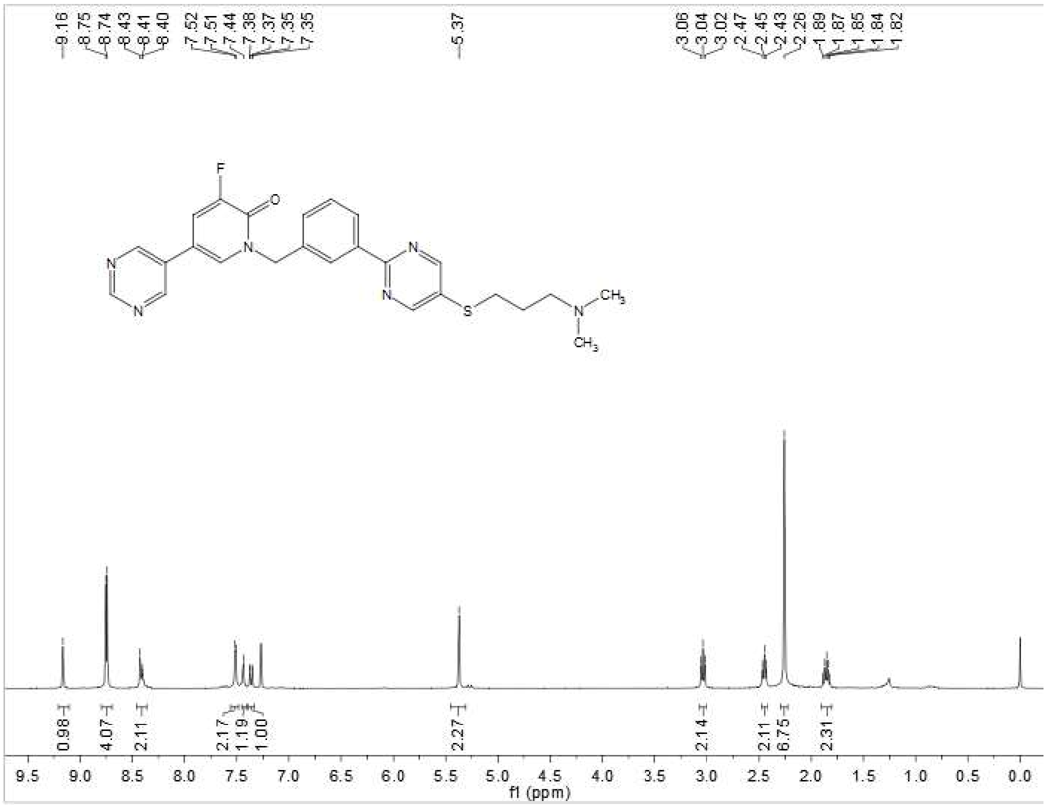

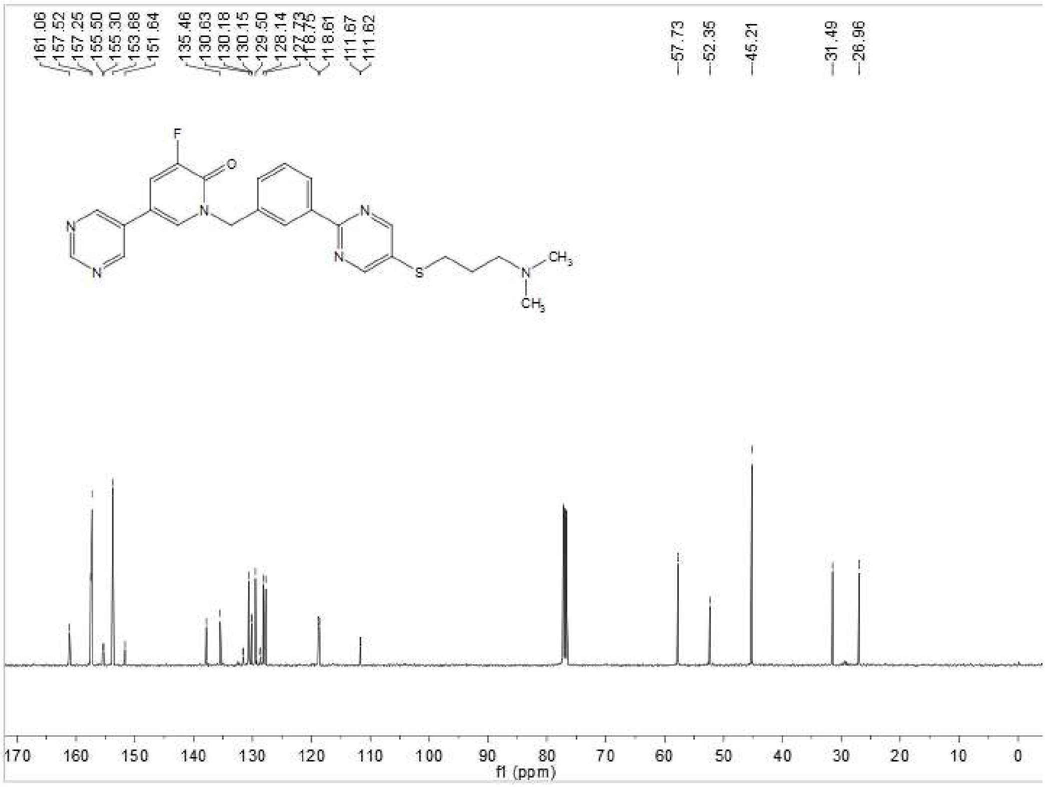

39

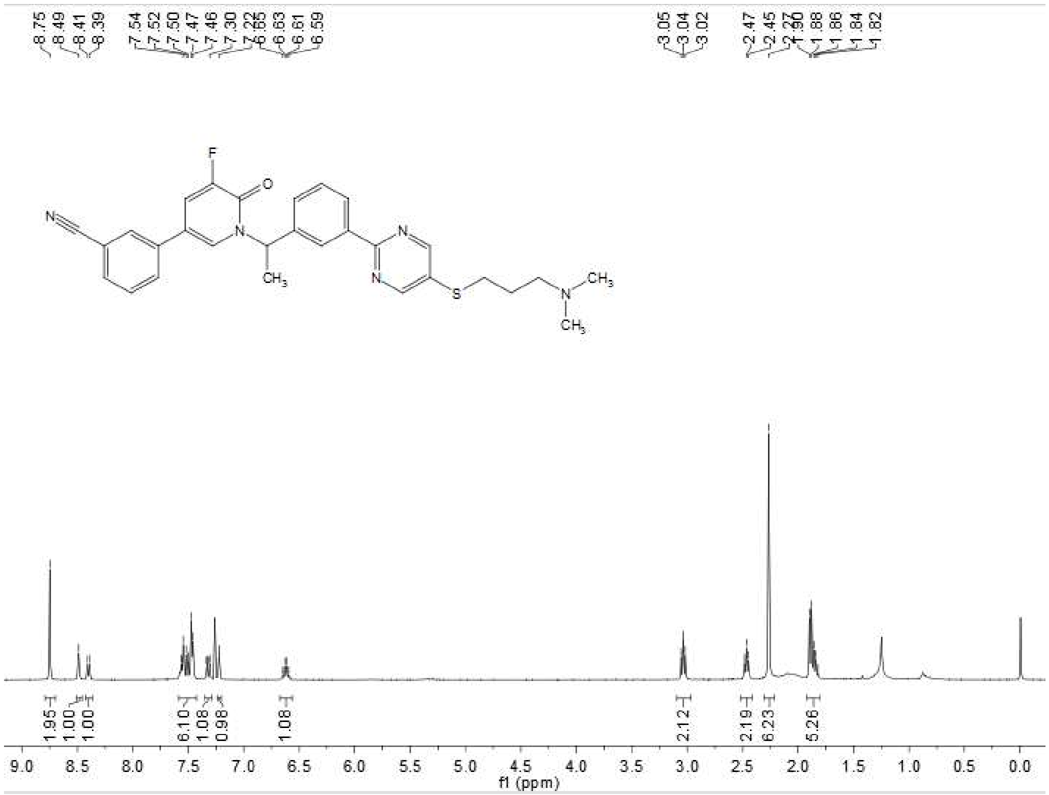

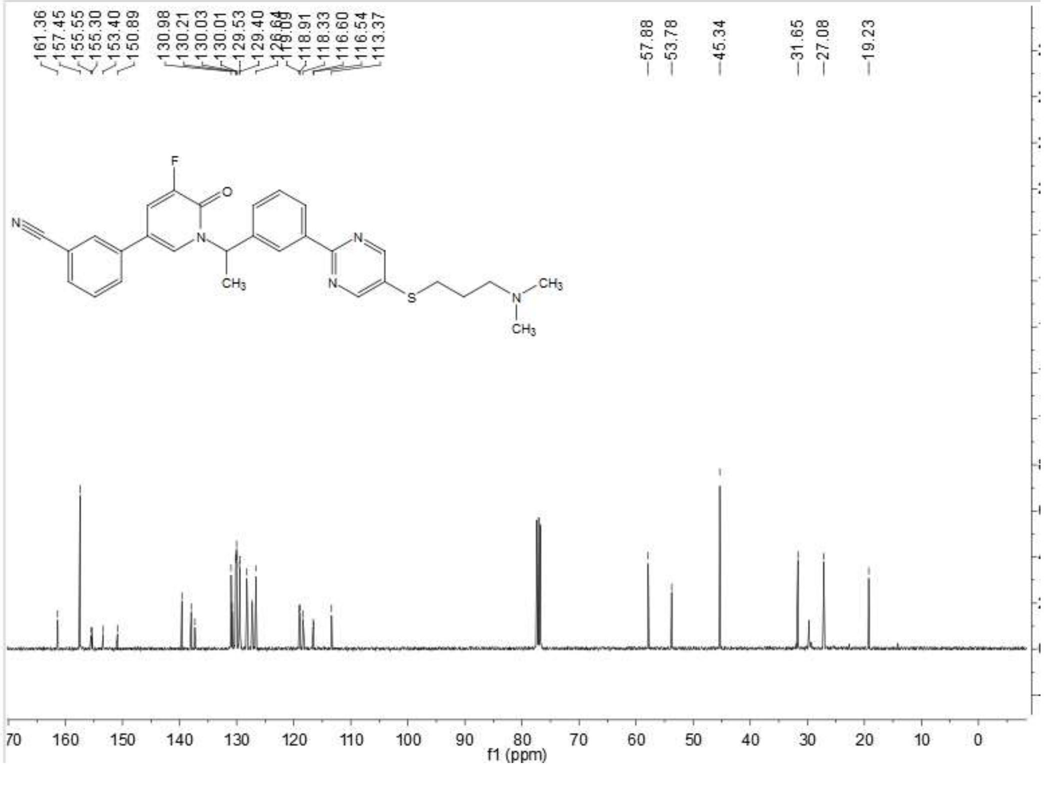

The chromatogram of the (*Rac*)-**31e**

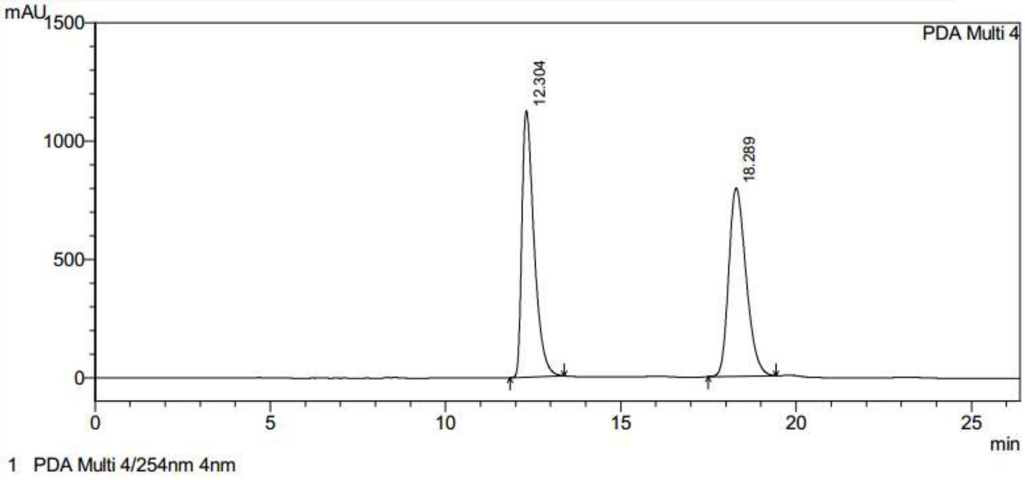

PeakTable

PDA Ch4 254nm 4nm

Peak#	Ret. Time	Area	Height	Area %	Height %
1	12.304	27166011	1127847	49.806	58.626
2	18.289	27377617	795944	50.194	41.374
Total		54543628	1923790	100.000	100.000

The chromatogram of the (*R*)-**31e** configuration (ee = 98.546%)

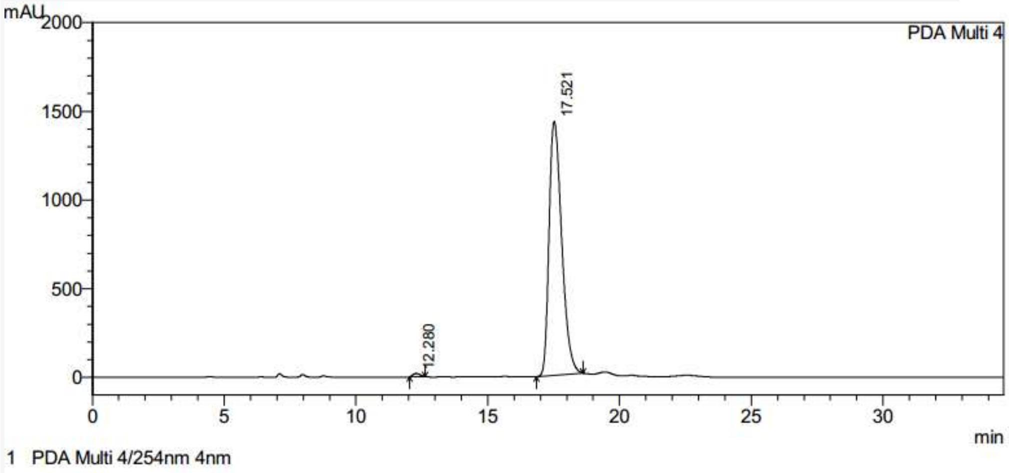

PeakTable

PDA Ch4 254nm 4nm

Peak#	Ret. Time	Area	Height	Area %	Height %
1	12.280	349597	18845	0.727	1.299
2	17.521	47765579	1431461	99.273	98.701
Total		48115176	1450306	100.000	100.000

The chromatogram of the (*S*)-**31e** configuration (ee = 94.824%)

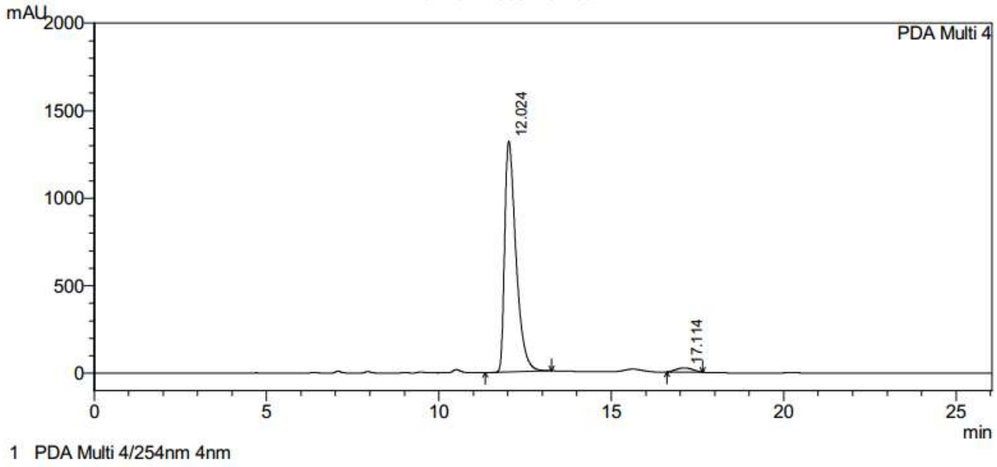

PeakTable

PDA Ch4 254nm 4nm

Peak#	Ret. Time	Area	Height	Area %	Height %
1	12.024	30103025	1317935	97.412	98.223
2	17.114	799694	23844	2.588	1.777
Total		30902719	1341779	100.000	100.000
